# Novel Triazole Hybrids of Betulin: Synthesis and Biological Activity Profile

**DOI:** 10.3390/molecules22111876

**Published:** 2017-11-01

**Authors:** Ewa Bębenek, Maria Jastrzębska, Monika Kadela-Tomanek, Elwira Chrobak, Beata Orzechowska, Katarzyna Zwolińska, Małgorzata Latocha, Anna Mertas, Zenon Czuba, Stanisław Boryczka

**Affiliations:** 1 Department of Organic Chemistry, School of Pharmacy with the Division of Laboratory Medicine in Sosnowiec, Medical University of Silesia in Katowice, 4 Jagiellońska Str., 41-200 Sosnowiec, Poland; mkadela@sum.edu.pl (M.K.-T.); echrobak@sum.edu.pl (E.C.); boryczka@sum.edu.pl (S.B.); 2 Department of Solid State Physics, Institute of Physics, University of Silesia, 4 Uniwersytecka Str., 40-007 Katowice, Poland; maria.jastrzebska@us.edu.pl; 3 Silesian Center for Education and Interdisciplinary Research, University of Silesia, 75 Pułku Piechoty 1, 41-500 Chorzów, Poland; 4 Hirszfeld Institute of Immunology and Experimental Therapy, Polish Academy of Sciences, Laboratory of Virology, 12 Rudolfa Weigla Str., 53-114 Wrocław, Poland; orzechow@iitd.pan.wroc.pl (B.O.); kjzwolinska@iitd.pan.wroc.pl (K.Z.); 5 Department of Cell Biology, School of Pharmacy with the Division of Laboratory Medicine in Sosnowiec, Medical University of Silesia in Katowice, 8 Jedności Str., 41-200 Sosnowiec; Poland; mlatocha@sum.edu.pl; 6 Department of Microbiology and Immunology, School of Medicine with the Division of Dentistry in Zabrze, Medical University of Silesia in Katowice, 19 Jordana Str., 41-808 Zabrze, Poland; amertas@sum.edu.pl (A.M.); zczuba@sum.edu.pl (Z.C.)

**Keywords:** betulin, 1,3-dipolar cycloaddition, triazoles, antiviral activity, anticancer activity, antimicrobial activity

## Abstract

Betulin derivatives containing a 1,2,3-triazole ring possess a wide spectrum of biological activities, including antiviral, anticancer, and antibacterial activity. A series of novel triazoles were prepared by the 1,3-dipolar cycloaddition reaction between the alkyne derivatives of betulin and organic azides. The chemical structures of the obtained compounds were defined by ^1^H and ^13^C NMR, IR, and high-resolution mass spectrometry (HR-MS) analysis. The target triazoles were screened for their antiviral activity against DNA and RNA viruses. The cytotoxic activity of the obtained compounds **5a**–**k** and **6a**–**h** was determined using five human cancer cell lines (T47D, MCF-7, SNB-19, Colo-829, and C-32) by a WST-1 assay. The bistriazole **6b** displayed a promising IC_50_ value (0.05 μM) against the human ductal carcinoma T47D (500-fold higher potency than cisplatin). The microdilution method was applied for an evaluation of the antimicrobial activity of all of the compounds. The triazole **5e** containing a 3′-deoxythymidine-5′-yl moiety exhibited antibacterial activity against two gram-negative bacteria vz. *Klebsiella pneumoniae* and *Escherichia coli* (minimal inhibitory concentration (MIC) range of 0.95–1.95 μM).

## 1. Introduction

The acetylenic moiety is one of the most important functional groups in organic chemistry [1]. Compounds with a carbon–carbon triple bond can be converted to triazole derivatives in the 1,3-dipolar cycloaddition reaction. The classic cycloaddition described by Huisgen was carried out at a high temperature and led to the formation of a mixture of 1,4- and 1,5-disubstituted 1,2,3-triazoles. In 2002, Sharpless and Meldal reported milder conditions of the 1,3-dipolar cycloaddition, which allowed for obtaining the 1,4-regioisomer with a high yield. The compounds bearing a 1,4-disubstituted 1,2,3-triazole ring were prepared by copper-catalyzed reactions of azides with terminal alkynes. The 1,3-dipolar cycloaddition was performed in various solvents using copper salts as a catalyst, giving 1,4-disubstituted products in high yields [2,3,4,5].

The 1,2,3-triazole ring is an attractive structural unit, which exhibits a high stability under acid/base hydrolysis conditions. Moreover, triazoles are capable of forming hydrogen bonds, which can be important for their bioavailability and solubility [6]. Several 1,2,3-triazoles (e.g., fluconazole, itraconazole, voriconazole and posaconazole) were used in medicine as antifungal drugs [7]. Additionally, the 1,2,3-triazoles are a class of heterocyclic compounds that possess a broad spectrum of biological activities, such as antibacterial [8,9,10], anticancer [11,12], antiviral [13,14], antiallergic [15], antitubercular [16,17,18], and anti-inflammatory [19,20] activity.

The triazoles can be used as a biological linker, and exhibit bioisosteric effects on aromatic rings, double bonds, peptide linkage and imidazole rings [21].

**Figure 1:**
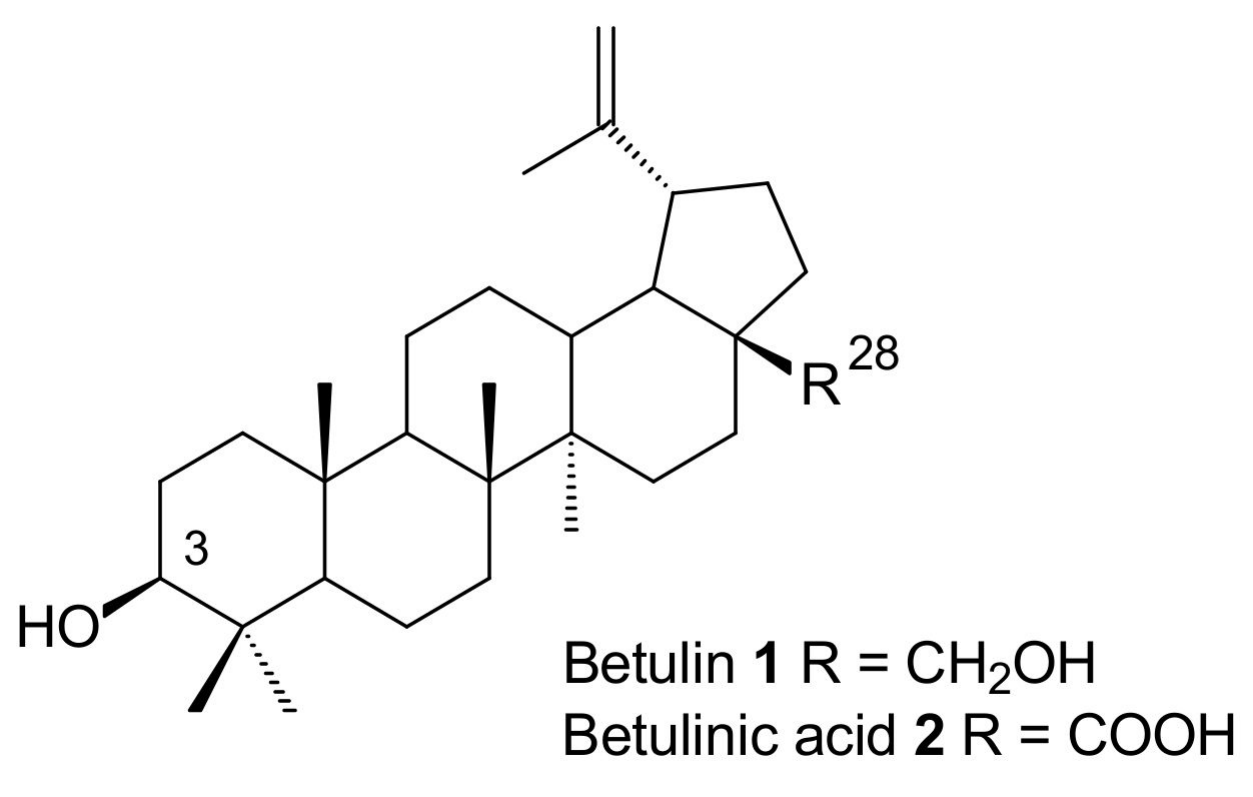
Chemical structures of betulin **1** and betulinic acid **2**.

Betulin **1** and betulinic acid **2** (Figure 1), both naturally occurring substances, were used in the synthesis of various derivatives containing an acetylenic moiety at the C-28 or C-3 positions. The 1,3-dipolar cycloaddition between acetylenic derivatives of triterpenes **1**–**2** and an azide generates substituted 1,2,3-triazoles showing anticancer, antiviral, and anti-inflammatory activities [22,23,24,25,26,27]. Novel L-ascorbic acid-conjugated pentacyclic triterpene derivatives connected via a triazole linker showed a potential anti-influenza activity against A/WSN/33 virus in MDCK (Madin Darby canine kidney) cells [28]. The conjugates of betulin **1** and betulinic acid **2** with 3′-azido-3′-deoxythymidine (AZT) prepared via the 1,3-dipolar cycloaddition exhibited a high anti-HIV activity. In addition, it is known that the triazole linkage is stable inside cells, and can interact with viral proteins [26].

The aim of the present work was to carry out the 1,3-dipolar cycloaddition reaction between acetylenic esters of betulin containing a propynoyl group at the C-28 and/or C-3 position, and organic azides. The antiviral activity of the new hybrid compounds was evaluated using a vesicular stomatitis virus (VSV), herpes simplex virus type-1 (HSV-1), cytopathogenic bovine orphan virus (ECBO), and human adenovirus type-5 (HAdV-5). The obtained triazoles were tested for their cytotoxic activity in vitro towards the T47D (human ductal carcinoma), MCF-7 (human adenocarcinoma), SNB-19 (glioblastoma), Colo-829 (human malignant melanoma), and C-32 (human amelanotic melanoma). Additionally, all of the compounds were investigated against the following microorganisms: *Staphylococcus aureus*, *Enterococcus faecalis*, *Escherichia coli*, *Pseudomonas aeruginosa*, *Klebsiella pneumoniae*, and *Candida albicans,* which are the most important nosocomial pathogens.

## 2. Results and Discussion

### 2.1. Chemistry

**Scheme 1:**
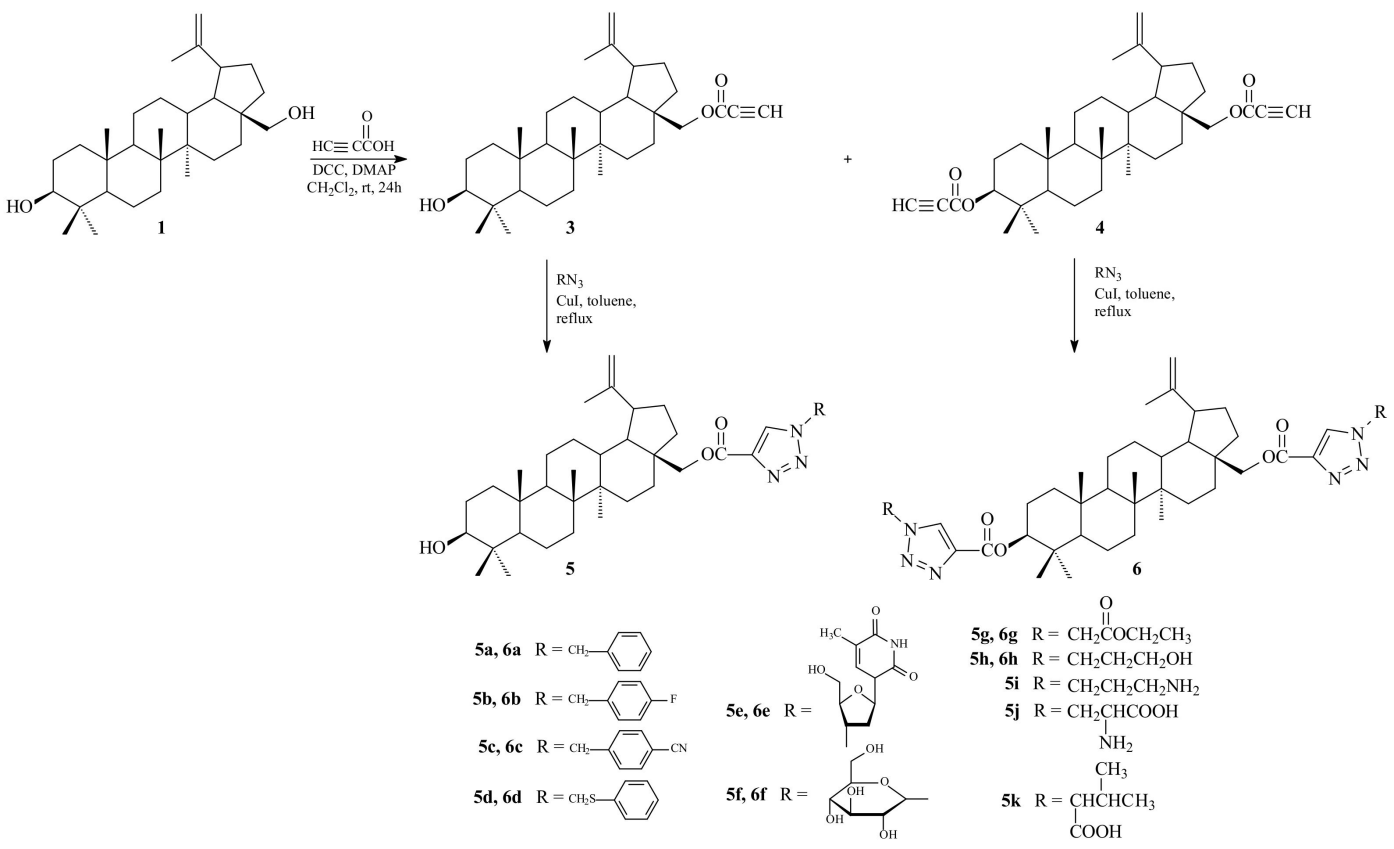
Synthesis of the acetylenic **3**–**4** and triazole derivatives of betulin **5a**–**k** and **6a**–**h**.

The new triazole hybrids of betulin were synthesized using 28-*O*-propynoylbetulin **3** and 3,28-*O,O’*-di(propynoyl)betulin **4** as starting compounds. The derivatives **3**–**4** were created in the reaction between betulin **1** and propiolic acid in the Steglich esterification (Scheme 1). The crude mixture of mono- and diester **4** was purified by column chromatography to give pure compounds **3** and **4** with 52% and 15% yields, respectively. The chemical structures of the obtained acetylenic derivatives **3**–**4** were determined by ^1^H, ^13^C, NMR, IR, and mass spectrometry (MS) spectra. The spectral data of **3** and **4** were consistent with those published in the literature [29].

**Figure 2:**
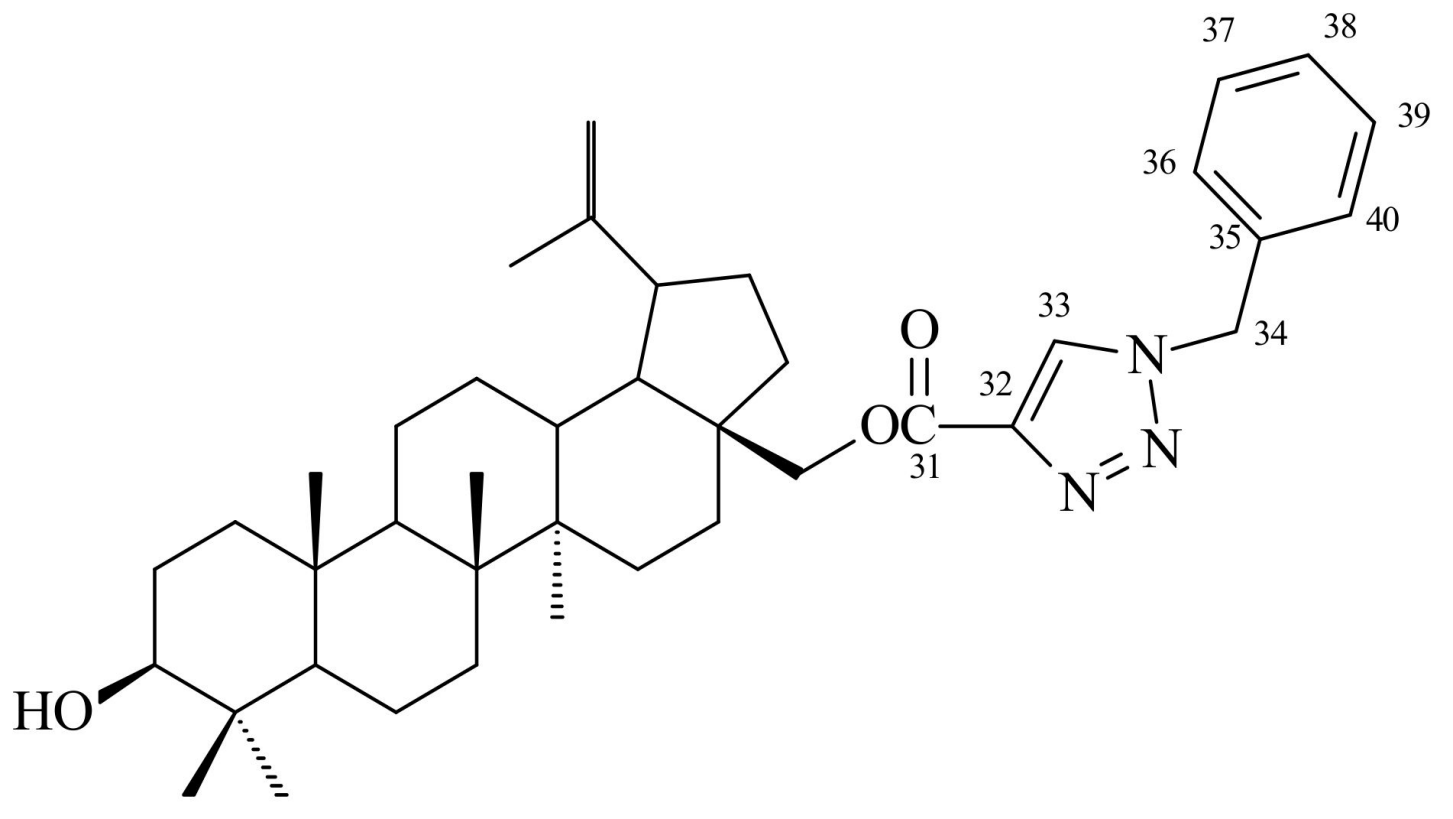
Chemical structure of triazole **5a**.

Treatment of 28-*O*-propynoylbetulin **3** with organic azides in toluene in the presence of copper iodide (I) under reflux gave substituted 1,2,3-triazoles. The major products of the reactions were separated by column chromatography to give pure derivatives **5a**–**k** in 44–78% yields. The chemical structures of the triazoles **5a**–**k** were defined by ^1^H NMR, ^13^C NMR and IR spectroscopy as well as high-resolution mass spectrometry (HR-MS) analysis. On the basis of previous studies, it occurred that the reaction of acetylenic derivatives with organic azides can lead to 1,4- or 1,5-regioisomeric products. For this reason, the 2D NMR (^1^H-^13^C HSQC and ^1^H-^13^C HMBC) spectra of compound **5a** were carried out (Figure 2, Table 1, Figure S1). For triazole **5a**, protons of the benzylic group (H-34) interacted with C-35 aromatic carbon. The presence of the 1,4-disubstituted 1,2,3-triazole ring was detected by the correlation of triazolyl proton H-33 with carbons C-32 and C-34.

The bistriazole derivatives of **6** were prepared in the reactions of the compound **4** with corresponding organic azides using the same conditions as those for compounds **5**. After purification by column chromatography, derivatives **6a**–**h** were received in 35–95% yields. The chemical structures of compounds **6a**–**h** were confirmed based on the ^1^H and ^13^C NMR, IR, and HR-MS spectra.

### 2.2. Antiviral Activity

#### 2.2.1. Characterization of Antiviral Activity against Viruses Belonging to Different Taxonomic Groups

To characterize the antiviral activity against VSV, HSV-1, ECBO, and HAdV-5, various experimental approaches were applied to the tested compounds. At first, the compounds were screened for their cytotoxic activity against the human lung adenocarcinoma epithelial (A549) and the human ovarian cancer (SKOV-3) cells using a two-fold dilution series of the tested substance ranging from 100 μg/mL to 0.5 μg/mL. For the antiviral activity assay, a CC_20_ (non-toxic concentration) was used for each compound. When the virucidal activity was assessed, none of the derivatives proved to be effective against the tested viruses. Similarly, in the pretreatment assay, none of the compounds protected the A549 cell against the same viruses. However, the results demonstrated that the compounds showed an inhibitory effect on ECBO and HAdV-5 added after the adsorption period. The acetylenic derivatives **3** and **4** did not exhibit antiviral activity against tested RNA and DNA viruses. Among the compounds assayed, six exhibited the selectivity index (SI; ratio of CC_50_ to EC_50_) ranging from 10 to 119 for ECBO (Table 2).

However, only two of them, **5h** and **6h**, had SI values greater than the betulin **1** (SI = 37). Bistriazole **6h** showed the highest antiviral activity, with an EC_50_ value of 0.25 μg/mL. Moreover, **6h** presented a SI of 119, which was approximately three times higher than the compound **1**′s value, and 11 times higher than the SI for ribavirin (Table 2). Comparing the analyzed compounds, only one, **5e**, exhibited a slight anti-HadV-5 activity with the SI = 6, which was below the SI of betulin **1** (data not shown). Based on the results of this work, one may conclude that the introduction of a polar hydroxy group (compounds **5h** and **6h**) in the triazole ring was advantageous for antiviral activity against ECBO virus. The introduction of a second triazole ring increased the antiviral activity against EBCO virus. In addition, triazole **5e**, which contains the pharmacophoric moiety (3′-azido-3′-deoxythymidine), shows a good antiviral activity toward the screening viruses.

The activity of bistriazole **6h** appeared to be Picornaviridae-specific (ECBO–Enteric, strain LCR-4), and this compound was essentially inactive against the negative-stranded RNA vesicular stomatitis virus (VSV), the double-stranded DNA herpes simplex virus type-1 (HSV-1) and human adenovirus type-5 (HAdV-5).

#### 2.2.2. Antiproliferative Effect of Triazole Derivatives

The antiproliferative activity of the compounds on the growth of human ovarian cancer cell line SKOV-3 in vitro was summarized in Table 3.

The tested compounds did not significantly inhibit the growth of the A549 cell line. However, several tested compounds inhibited the proliferation of ovarian cancer cells (SKOV-3) in a dose-dependent manner, with IC values of 10–50 μg/mL (IC is the concentrations of compounds that where used to inhibit the proliferation of tumor cell line) The compounds **5e** and **6h** had the highest antiproliferative activity, reducing the proliferation of the SKOV-3 cell by 75% and 70% respectively, at 50 μg/mL (Table 3). Betulin **1** showed an almost comparable activity with respect to cisplatin, reducing the proliferation of SKOV-3 by 30% at 2.5 μg/mL. The optimal concentration of compounds **5e** and **6h** required to achieve such a dramatic effect on the cell proliferation is higher than that of betulin **1** (50 μg/mL). Nevertheless, it is noteworthy that these compounds displayed a lower toxicity than the betulin **1**.

### 2.3. Anticancer Activity

The triazoles **5a**–**k** and bistriazoles **6a**–**h** were tested for their cytotoxic activity against human cancer cell lines such as: T47D (human ductal carcinoma), MCF-7 (human adenocarcinoma), SNB-19 (glioblastoma), Colo-829 (human malignant melanoma), and C-32 (human amelanotic melanoma). The WST-1 assay is used to evaluate the cytotoxic activity of triterpene derivatives. The cytotoxic activity data were expressed as the concentration of compounds (μM), which inhibits the proliferation of 50% of tumor cells as compared with the control untreated cells. Betulin **1**, acetylenic derivatives **3**–**4**, and cisplatin were used as a positive control. The resulting IC_50_ values are summarized in Table 4. In the present work, two hormone-dependent breast cancer lines (T47D and MCF-7) have been used, which are often applied for the analyses of breast cancer mechanisms. The performed studies suggested that in cells T47D (caspase-3 positive) and MCF-7 (caspase-3 negative), another mechanism of DNA fragmentation was observed [30].

Orchel et al. observed that betulin **1** and 28-*O*-propynoylbetulin **3** induced apoptosis in G-361 cells e.g., via caspase-3 activation [31]. The tested compounds were the most active towards the human breast cancer cells T47D (caspase-3 positive) compared with MCF-7 cells. The highest cytotoxic activity against T47D cells in the group of monotriazoles was exhibited by the derivative **5h** (IC_50_ value 1.3 μM), which was 19 time more cytotoxic than cisplatin. In the case of triazoles **5e**, **5f**, and **5j**, which contain 3′-deoxythymidine-5′-yl, 1-deoxy-β-d-glucopyranosyl, and 2-aminocarboxypropyl substituents, respectively, the total decay of cytotoxic activity against the T47D cells was noted. The bistriazole **6b**, which contains two fluorine moieties, showed a good cytotoxic activity against T47D, MCF-7, and SNB-19 (IC_50_ = 0.05 μM, 0.09 μM, and 0.08 μM, respectively). Additionally, a lower cytotoxic activity of the tested compounds against the C-32 cell line was observed.

### 2.4. Antimicrobial Activity

The antibacterial activity of triterpene derivatives (betulin **1**, **3**–**4**, **5a**–**k**, and **6a**–**h**) was evaluated using gram-positive (*Staphylococcus aureus* ATCC 25923, *Enterococcus faecalis* ATCC 29212) and gram-negative (*Escherichia coli* ATTC 25922, *Pseudomonas aeruginosa* ATTC 27853, *Klebsiella pneumoniae* ATTC 700603) bacteria. Additionally, the antifungal activity of the tested compounds was investigated using the *Candida albicans* ATTC 10231 strain. The minimal inhibitory concentration (MIC) and minimal bactericidal concentration (MBC) were determined by the broth microdilution method, according to the Clinical and Laboratory Standards Institute [32,33].

Various triterpene derivatives collected from the plant species were studied as antimicrobial agents. Ursolic and oleanic acids isolated from the Miconia species exhibited an antibacterial activity against the following microorganisms: *Streptococcus mitis*, *Streptococcus sanguinis*, *Streptococcus mutans*, *Streptococcus salivarius*, *Streptococcus sorbinus,* and *Enterococcus faecalis*. The MIC values of these triterpene acids towards *Enterococcus faecalis* ranged from 40–50 μg/mL [34].

In the applied microdilution assay, only the **5e** compounds showed an antibacterial activity against *Klebsiella pneumoniae* and *Escherichia coli*, with MIC values of 0.95 μM and 1.95 μM, respectively (Table 5). The compound **5e** exhibited low MBC:MIC ratios of ≥4 for two gram-negative bacteria strains. The MCB:MIC ratio is a major parameter that reflects the bactericidal capacity of the tested compounds. A value superior to one in the MBC:MIC ratio suggests that a great amount of compound is needed to reach the bactericidal activity [35]. The MBC:MIC ratio is no more than four, which suggests that triazole **5e** possess the bactericidal activity against *Escherichia coli*. The results of study showed that compound **5e** is bacteriostatic against the *Klebsiella pneumoniae strain*, with a MBC:MIC ratio of >4.

## 3. Materials and Methods

### 3.1. General Techniques

Melting points were determined in the open capillary tubes on an Electrothermal IA 9300 melting point apparatus Electrothermal Engineering Ltd, Rochford, UK. The values given are uncorrected. Optical rotation properties were measured with an ATAGO SAC-I polarimeter (Atago, Tokyo, Japan) using a sodium lamp (589 nm) at 20 °C. The NMR spectra (600/150 MHz) were recorded on a Bruker Avance (Bruker , Billerica, MA, USA) III 600 spectrometer in CDCl_3_. Chemical shifts were reported in ppm (δ), and J values in Hz. Multiplicity was designated as the singlet (s), doublet (d), triplet (t), quartet (q) and multiplet (m). High-resolution mass spectral (HR-MS) analysis was performed on a Bruker Impact II instrument. Solid state infrared spectra were recorded in the range of 4000–1000 cm^−1^ using the Shimadzu IRAffinity-1 FTIR spectrometer (Shimadzu, Kyoto, Japan) and KBr pellet method. Thin layer chromatography (TLC) was performed on silica gel 60 254F plates (Merck, Darmstadt, Germany) using a mixture of different organic solvents as an eluent. The chromatographic spots were detected by spraying with a solution of 5% sulfuric acid, followed by heating. Column chromatography was performed on silica gel 60, <63 μm (Merck), with the mixture of chloroform and ethanol (15:1, *v*/*v*) or hexane and ethyl acetate (3:2, *v*/*v*) as an eluent. Solvents were dried and purified according to usual procedures.

### 3.2. Synthesis of 28-O-Propynoylbetulin ***3*** and 3,28-O,O’-di(propynoyl)betulin ***4***

28-*O*-Propynoylbetulin **3** (m.p. 132–134, lit. [29] m.p. 133–135 °C) and 3,28-*O*,*O*’-di(propynoyl)betulin **4** (m.p. 101–103, lit. [29] m.p. 102–104 °C) were synthesized according to the method described previously [29]. The spectral data of **3** and **4** were consistent with those published in the literature [29].

### 3.3. General Procedure for the Synthesis of Triazole Derivatives of Betulin ***5***

To a solution of 28-*O*-propynoylbetulin **3** (0.1 g, 0.203 mmol) and copper iodide (I) (0.1 eqv, 0.004 g, 0.020 mmol) in dry toluene (4 mL), a corresponding organic azide (1.05 eqv, 0.213 mmol) was added. The reaction mixture was stirred under reflux for 72 h. Then, the reaction mixture was concentrated under reduced pressure. The crude products were purified by column chromatography using various mixtures of organic solvents to give pure triazoles **5a**–**k**:

*28-O-(1-Benzyl-1H-1,2,3-triazol-4-yl)carbonylbetulin* (**5a**). Yield: 66%, m.p. 169–171 °C, ${\left[\alpha \right]}_{D}^{20}$ −0.5 (*c* 1, CHCl_3_). ^1^H NMR (600 MHz, CDCl_3_) δ: 0.78 (s, 3H, CH_3_), 0.84 (s, 3H, CH_3_), 0.98 (s, 3H, CH_3_), 1.00 (s, 3H, CH_3_), 1.06 (s, 3H, CH_3_), 1.67 (s, 3H, CH_3_), 1.10–2.09 (m, 25H, CH, CH_2_), 2.51 (m, 1H, H-19), 3.19 (m, 1H, H-3), 4.14 (d, *J* = 10.8 Hz, 1H, H-28), 4.56 (d, *J* = 10.8 Hz, 1H, H-28), 4.61 (s, 1H, H-29), 4.71 (s, 1H, H-29), 5.60 (s, 2H, CH_2_), 7.31–7.32 (m, 2H, H_Ar_), 7.41–7.42 (m, 3H, H_Ar_), 7.97 (s, 1H, CH-triazol). ^13^C NMR (150 MHz, CDCl_3_) δ: 14.8, 15.4, 16.0, 16.1, 18.3, 19.1, 20.8, 22.7, 25.2, 27.1, 27.4, 27.9, 29.6, 29.8, 31.9, 34.2, 34.7, 37.2, 37.7, 38.7, 38.9, 40.9, 42.7, 46.7, 47.7, 48.9, 50.4, 54.5, 55.3, 63.6, 78.9, 109.9, 127.1, 128.2, 129.2, 129.3, 133.8, 140.6, 150.1, 161.2. IR (KBr, cm^−1^) ν_max_: 1246–1147 (N-N=N), 1457 (N=N), 1527 (C=N), 1731 (C=O). HR-MS (APCI) *m*/*z*: C_40_H_57_N_3_O_3_ [(M − H)^−^], Calc. 626.4322; Found 626.4325 (Figure S2).

*28-O-[1-(4-Fluorobenzyl)-1H-1,2,3-triazol-4-yl]carbonylbetulin* (**5b**). Yield: 49%, m.p. 144–147 °C, ${\left[\alpha \right]}_{D}^{20}$ −0.6 (*c* 1, CHCl_3_). ^1^H NMR (600 MHz, CDCl_3_) δ: 0.78 (s, 3H, CH_3_), 0.86 (s, 3H, CH_3_), 0.98 (s, 3H, CH_3_), 1.00 (s, 3H, CH_3_), 1.06 (s, 3H, CH_3_), 1.67 (s, 3H, CH_3_), 1.11–2.10 (m, 25H, CH, CH_2_), 2.52 (m, 1H, H-19), 3.21 (m, 1H, H-3), 4.15 (d, *J* = 10.8 Hz, 1H, H-28), 4.56 (d, *J* = 10.8 Hz, 1H, H-28), 4.62 (s, 1H, H-29), 4.72 (s, 1H, H-29), 5.57 (s, 2H, CH_2_), 7.10–7.13 (m, 2H, H_Ar_), 7.31–7.33 (m, 2H, H_Ar_), 7.97 (s, 1H, CH-triazol). ^13^C NMR (150 MHz, CDCl_3_) δ: 14.8, 15.4, 16.0, 16.1, 18.3, 19.1, 20.8, 25.2, 27.1, 27.4, 27.9, 29.6, 29.8, 34.2, 34.7, 37.2, 37.7, 38.7, 38.9, 40.9, 42.7, 46.7, 47.7, 48.9, 50.4, 53.7, 55.3, 63.6, 78.9, 109.9, 116.3, 116.5, 126.9, 129.7, 130.1, 130.2, 140.7, 150.1, 161.1, 162.2, 163.9. IR (KBr, cm^−1^) ν_max_: 822 (C-F), 1226–1147 (N-N=N), 1512 (N=N), 1608 (C=N), 1729 (C=O). HR-MS (APCI) *m*/*z*: C_40_H_56_FN_3_O_3_ [(M − H)^−^], Calc. 644.4227; Found 644.4229 (Figure S3).

*28-O-[1-(4-Cyanobenzyl)-1H-1,2,3-triazol-4-yl]carbonylbetulin* (**5c**). Yield: 47%, m.p. 225–228 °C, ${\left[\alpha \right]}_{D}^{20}$ +0.3 (*c* 1, CHCl_3_). ^1^H NMR (600 MHz, CDCl_3_) δ: 0.78 (s, 3H, CH_3_), 0.85 (s, 3H, CH_3_), 0.99 (s, 3H, CH_3_), 1.01 (s, 3H, CH_3_), 1.07 (s, 3H, CH_3_), 1.68 (s, 3H, CH_3_), 1.12–2.10 (m, 25H, CH, CH_2_), 2.51 (m, 1H, H-19), 3.20 (m, 1H, H-3), 4.15 (d, *J* = 10.8 Hz, 1H, H-28), 4.58 (d, *J* = 10.8 Hz, 1H, H-28), 4.62 (s, 1H, H-29), 4.72 (s, 1H, H-29), 5.68 (s, 2H, CH_2_), 7.40 (d, 2H, *J* = 8.4 Hz, H_Ar_), 7.72 (d, 2H, *J* = 8.4 Hz, H_Ar_), 8.05 (s, 1H, CH-triazol). ^13^C NMR (150 MHz, CDCl_3_) δ: 14.8, 15.4, 16.0, 16.1, 18.3, 19.1, 20.8, 21.1, 25.2, 27.1, 27.4, 27.9, 29.6, 29.8, 34.2, 34.7, 37.2, 37.7, 38.7, 38.9, 40.9, 42.7, 46.7, 47.7, 48.9, 50.4, 53.7, 55.3, 60.4, 63.8, 78.9, 109.9, 113.3, 117.9, 127.3, 128.5, 133.1, 138.9, 141.0, 150.0, 160.9. IR (KBr, cm^−1^) ν_max_: 1246–1147 (N-N=N), 1457 (N=N), 1612 (C=N), 1726 (C=O), 2232 (CN). HR-MS (APCI) *m*/*z*: C_41_H_56_N_4_O_3_ [(M − H)^−^], Calc. 651.4274; Found 651.4276 (Figure S4).

*28-O-(1-Phenylthiomethyl-1H-1,2,3-triazol-4-yl)carbonylbetulin* (**5d**). Yield 78%, m.p. 143–146 °C, ${\left[\alpha \right]}_{D}^{20}$ −0.6 (*c* 1, CHCl_3_). ^1^H NMR (600 MHz, CDCl_3_) δ: 0.79 (s, 3H, CH_3_), 0.85 (s, 3H, CH_3_), 0.99 (s, 3H, CH_3_), 1.02 (s, 3H, CH_3_), 1.07 (s, 3H, CH_3_), 1.67 (s, 3H, CH_3_), 1.14–2.10 (m, 25H, CH, CH_2_), 2.51 (m, 1H, H-19), 3.22 (m, 1H, H-3), 4.15 (d, *J* = 10.8 Hz, 1H, H-28), 4.56 (d, *J* = 10.8 Hz, 1H, H-28), 4.56 (s, 1H, H-29), 4.73 (s, 1H, H-29), 5.69 (s, 2H, CH_2_), 7.35–7.36 (m, 5H, H_Ar_), 8.05 (s, 1H, CH-triazol). ^13^C NMR (150 MHz, CDCl_3_) δ: 14.1, 14.8, 15.4, 16.0, 16.1, 18.3, 19.1, 20.8, 22.7, 25.2, 27.1, 27.4, 27.9, 29.6, 29.8, 34.2, 34.7, 37.2, 37.7, 38.7, 38.9, 40.9, 42.8, 46.7, 47.7, 48.9, 50.4, 54.3, 55.3, 63.6, 78.9, 109.9, 126.8, 129.1, 129.7, 131.4, 132.4, 140.6, 150.0, 160.9. IR (KBr, cm^−1^) ν_max_: 1243–1136 (N-N=N), 1482 (N=N), 1546 (C=N), 1730 (C=O). HR-MS (APCI) *m*/*z*: C_40_H_57_N_3_O_3_S [(M − H)^−^], Calc. 658.4043; Found 658.4041 (Figure S5).

*28-O-[1-(3′-Deoxythymidine-5′-yl)-1H-1,2,3-triazol-4-yl]carbonylbetulin* (**5e**). Yield 67%, m.p. 181–185 °C, ${\left[\alpha \right]}_{D}^{20}$ +0.7 (*c* 1, EtOH). ^1^H NMR (600 MHz, DMSO-*d*_6_) δ: 0.66 (s, 3H, CH_3_), 0.78 (s, 3H, CH_3_), 0.88 (s, 3H, CH_3_), 0.97 (s, 3H, CH_3_), 1.03 (s, 3H, CH_3_), 1.67 (s, 3H, CH_3_), 1.78 (s, 3H, CH_3_-AZT), 1.05–2.1.85 (m, 27H, CH, CH_2_), 2.00 (m, 1H, AZT), 2.67 (m, 1H, H-19), 2.77 (m, 1H, AZT), 2.97 (m, 1H, H-3), 3.65 (m, 2H, AZT), 4.02 (d, *J* = 10.8 Hz, 1H, H-28), 4.28 (t, *J* = 4.8 Hz, 1H, AZT), 4.54 (d, *J* = 10.8 Hz, 1H, H-28), 4.59 (s, 1H, H-29), 4.74 (s, 1H, H-29), 5.28 (t, 1H, *J* = 4.8 Hz, AZT), 5.46 (m, 1H, AZT), 5.67 (s, 1H, AZT), 6.45 (t, 1H, *J* = 6.6 Hz, AZT), 7.83 (s, 1H, AZT), 8.32 (s, 1H, CH-triazol), 9.02 (s, 2H, NH-AZT). ^13^C NMR (150 MHz, DMSO-*d*_6_) δ: 12.7, 15.0, 15.9, 16.1, 16.3, 16.4, 18.4, 19.2, 20.8, 25.2, 25.3, 25.6, 27.1, 27.6, 28.6, 29.4, 29.7, 34.2, 34.6, 36.6, 37.1, 37.6, 37.7, 38.7, 39.0, 40.9, 42.8, 46.8, 47.5, 48.7, 50.2, 55.3, 55.4, 55.6, 60.2, 60.6, 61.1, 61.3, 62.7, 66.8, 67.5, 77.3, 79.7, 83.8, 84.3, 84.4, 84.7, 110.0, 110.1, 110.5, 129.3, 136.5, 136.8, 139.3, 150.3, 150.9, 160.9, 164.2. IR (KBr, cm^−1^) ν_max_: 1273 (N-N=N), 1456 (N=N), 1541 (C=N), 1697 (C=O), 2104 (C-NH_2_), 2870–2941 (C-H), 3392–3412 (N-H). HR-MS (APCI) *m*/*z*: C_43_H_63_N_5_O_7_ [(M − H)^−^], Calc. 760.4649; Found 760.4645 (Figure S6).

*28-O-[1-(1-Deoxy-β-D-glucopyranosyl)-1H-1,2,3-triazol-4-yl]carbonylbetulin* (**5f**). Yield 75%, m.p. 184–186 °C, ${\left[\alpha \right]}_{D}^{20}$ +0.9 (*c* 1, EtOH). ^1^H NMR (600 MHz, DMSO-*d*_6_) δ: 0.66 (s, 3H, CH_3_), 0.78 (s, 3H, CH_3_), 0.85 (s, 3H, CH_3_), 0.97 (s, 3H, CH_3_), 1.05 (s, 3H, CH_3_), 1.67 (s, 3H, CH_3_), 1.11–1.72 (m, 25H, CH, CH_2_), 1.99 (m, 1H, OH), 2.98 (m, 1H, H-19), 3.26 (m, 1H, H-3), 3.41 (m, 1H, OH), 3.47 (m, 1H, OH), 3.71 (m, 1H, OH), 3.86 (m, 1H, CH-sugar), 4.02 (d, *J* = 10.8 Hz, 1H, H-28), 4.28 (m, 1H, CH-sugar), 4.56 (d, *J* = 10.8 Hz, 1H, H-28), 4.58 (s, 1H, H-29), 4.63 (m, 1H, CH-sugar), 4.73 (s, 1H, H-29), 5.20 (d, 1H, *J* = 5.4 Hz, CH-sugar), 5.34 (d, 1H, *J* = 5.4 Hz, CH-sugar), 5.45 (d, 1H, *J* = 5.4 Hz, CH-sugar), 5.61 (d, 1H, *J* = 5.4 Hz, CH-sugar), 9.08 (s, 1H, CH-triazol). ^13^C NMR (150 MHz, DMSO-*d*_6_) δ: 15.0, 16.1, 16.3, 16.4, 18.4, 19.2, 20.8, 25.2, 27.1, 27.6, 28.6, 29.4, 29.6, 34.2, 34.6, 37.1, 37.6, 38.7, 39.0, 39.6, 40.9, 42.8, 46.8, 47.5, 48.7, 50.2, 55.3, 61.2, 62.7, 69.9, 72.4, 77.2, 77.3, 79.7, 80.6, 88.3, 110.5, 129.1, 139.2, 150.3, 160.9. IR (KBr, cm^−1^) ν_max_: 1275 (N-N=N), 1456 (N=N), 1541 (C=N), 1728 (C=O), 2870–2941 (C-H), 3414 (O-H). HR-MS (APCI) *m*/*z*: C_39_H_61_N_3_O_8_ [(M − H)^−^], Calc. 698.4380; Found 698.4384.

*28-O-(1-Ethylacetyl-1H-1,2,3-triazol-4-yl)carbonylbetulin* (**5g**). Yield 65%, m.p. 176–181 °C, ${\left[\alpha \right]}_{D}^{20}$ −0.1 (*c* 1, CHCl_3_). ^1^H NMR (600 MHz, CDCl_3_) δ: 0.78 (s, 3H, CH_3_), 0.85 (s, 3H, CH_3_), 0.98 (s, 3H, CH_3_), 1.00 (s, 3H, CH_3_), 1.08 (s, 3H, CH_3_), 1.34 (t, 3H, *J* = 7.2 Hz, CH_3_), 1.69 (s, 3H, CH_3_), 1.18–2.00 (m, 25H, CH, CH_2_), 2.53 (m, 1H, H-19), 3.20 (m, 1H, H-3), 4.17 (d, *J* = 10.8 Hz, 1H, H-28), 4.32 (q, 2H, *J* = 7.2Hz, OCH_2_), 4.59 (d, *J* = 10.8 Hz, 1H, H-28), 4.63 (s, 1H, H-29), 4.73 (s, 1H, H-29), 5.24 (s, 2H, CH_2_), 8.24 (s, 1H, CH-triazol). ^13^C NMR (150 MHz, CDCl_3_) δ: 14.1, 14.8, 15.4, 16.1, 18.3, 19.2, 20.8, 25.21, 27.1, 27.4, 28.0, 29.6, 29.8, 34.2, 34.7, 37.2, 37.7, 38.7, 38.9, 40.9, 42.8, 46.7, 47.7, 48.9, 50.4, 51.0, 55.3, 62.8, 63.7, 79.0, 110.0, 128.7, 140.7, 150.1, 160.9, 165.7. IR (KBr, cm^−1^) ν_max_: 1213 (N-N=N), 1458 (N=N), 1541 (C=N), 1734 (C=O), 1751 (C=O), 2870–2943 (C-H). HR-MS (APCI) *m*/*z*: C_37_H_57_N_3_O_5_ [(M − H)^−^], Calc. 622.4172; Found 622.4170.

*28-O-[1-(3-Hydroxypropyl)-1H-1,2,3-triazol-4-yl]carbonylbetulin* (**5h**). Yield: 77%, m.p. 201–205 °C, ${\left[\alpha \right]}_{D}^{20}$ −1.5 (*c* 1, EtOH). ^1^H NMR (600 MHz, DMSO-*d*_6_) δ: 0.66 (s, 3H, CH_3_), 0.78 (s, 3H, CH_3_), 0.88 (s, 3H, CH_3_), 0.96 (s, 3H, CH_3_), 1.03 (s, 3H, CH_3_), 1.08 (t, 2H, *J* = 7.2 Hz, CH_2_OH), 1.67 (s, 3H, CH_3_), 1.13–1.74 (m, 25H, CH, CH_2_), 2.51 (m, 1H, H-19), 2.98 (m, 1H, H-3), 3.39 (m, 2H, CH_2_), 4.02 (d, *J* = 10.8 Hz, 1H, H-28), 4.30 (br.s, 1H, OH), 4.48 (t, 2H, *J* = 7.2 Hz, CH_2_), 4.54 (d, *J* = 10.8 Hz, 1H, H-28), 4.58 (s, 1H, H-29), 4.73 (s, 1H, H-29), 8.81 (s, 1H, CH-triazol). ^13^C NMR (150 MHz, , DMSO-*d*_6_) δ: 15.0, 15.9, 16.1, 16.4, 18.4, 19.2, 20.8, 25.2, 25.3, 27.1, 27.6, 28.6, 29.4, 29.7, 33.1, 34.2, 34.6, 37.1, 37.6, 38.7, 39.0, 40.9, 42.8, 46.8, 47.5, 47.6, 48.7, 50.2, 55.3, 55.6, 57.8, 62.5, 77.2, 79.7, 99.5, 110.5, 129.7, 139.0, 150.3, 161.1. IR (KBr, cm^−1^) ν_max_: 1201 (N-N=N), 1456 (N=N), 1541 (C=N), 1724 (C=O), 2870–2941 (C-H), 3420 (O-H). HR-MS (APCI) *m*/*z*: C_36_H_57_N_3_O_4_ [(M − H)^−^], Calc. 594.4271; Found 594.4274.

*28-O-[1-(3-Aminopropyl)-1H-1,2,3-triazol-4-yl]carbonylbetulin* (**5i**). Yield: 66%, m.p. 156–160 °C, ${\left[\alpha \right]}_{D}^{20}$ +1.9 (*c* 1, EtOH). ^1^H NMR (600 MHz, DMSO-*d*_6_) δ: 0.67 (s, 3H, CH_3_), 0.78 (s, 3H, CH_3_), 0.88 (s, 3H, CH_3_), 0.97 (s, 3H, CH_3_), 1.03 (s, 3H, CH_3_), 1.68 (s, 3H, CH_3_), 1.15–1.83 (m, 25H, CH, CH_2_), 1.97 (m, 2H, CH_2_), 2.58 (m, 1H, H-19), 2.60 (t, 2H, *J* = 7.2 Hz, CH_2_NH_2_), 2.98 (m, 1H, H-3), 4.00 (d, *J* = 10.8 Hz, 1H, H-28), 4.27 (br.s, 2H, NH_2_), 4.51 (t, 2H, *J* = 7.2 Hz, CH_2_), 4.55 (d, *J* = 10.8 Hz, 1H, H-28), 4.59 (s, 1H, H-29), 4.73 (s, 1H, H-29), 8.82 (s, 1H, CH-triazol). ^13^C NMR (150 MHz, DMSO-*d*_6_) δ: 15.0, 16.1, 16.3, 16.4, 18.4, 19.2, 20.8, 25.2, 27.1, 27.6, 28.6, 29.5, 29.7, 33.9, 34.2, 34.6, 37.2, 37.6, 38.1, 38.7, 38.9, 39.0, 40.9, 42.8, 46.8, 47.5, 47.9, 48.7, 50.2, 55.3, 62.6, 77.2, 110.5, 129.7, 139.1, 150.3, 161.1. IR (KBr, cm^−1^) ν_max_: 1205 (N-N=N), 1456 (N=N), 1541 (C=N), 1732 (C=O), 2870–2939 (C-H), 3385–3442 (N-H). HR-MS (APCI) *m*/*z*: C_36_H_58_N_4_O_3_ [(M − H)^−^], Calc. 593.4431; Found 593.4333.

*2-Amino-3-[4-(28-O-betulinylcarbonyl)-1H-1,2,3-triazol-1-yl]propanoic acid* (**5j**). Yield: 44%, m.p. 265–269 °C, ${\left[\alpha \right]}_{D}^{20}$ −1.1 (*c* 1, EtOH). ^1^H NMR (600 MHz, DMSO-*d*_6_) δ: 0.66 (s, 3H, CH_3_), 0.78 (s, 3H, CH_3_), 0.88 (s, 3H, CH_3_), 0.96 (s, 3H, CH_3_), 1.03 (s, 3H, CH_3_), 1.06 (t, 1H, *J =* 7.2 Hz, CH), 1.67 (s, 3H, CH_3_), 1.14–1.81 (m, 25H, CH, CH_2_), 1.97 (m, 2H, CH_2_), 2.58 (m, 1H, H-19), 2.96 (m, 1H, H-3), 3.97 (d, *J* = 10.8 Hz, 1H, H-28), 4.29 (d, 2H, *J* = 7.2 Hz, CH_2_), 4.55 (d, *J* = 10.8 Hz, 1H, H-28), 4.57 (s, 1H, H-29), 4.73 (s, 1H, H-29), 8.62 (s, 1H, CH-triazol). IR (KBr, cm^−1^) ν_max_: 1284 (N-N=N), 1456 (N=N), 1603 (C=N), 1728 (C=O), 2870–2938 (C-H), 3406 (O-H). HR-MS (APCI) *m*/*z*: C_36_H_56_N_4_O_5_ [(M − H)^−^], Calc. 623.4172; Found 623.4175.

*3-Methyl-3-[4-(28-O-betulinylcarbonyl)-1H-1,2,3-triazol-1-yl]butyric acid* (**5k**). Yield: 55%, m.p. 293–297 °C, ${\left[\alpha \right]}_{D}^{20}$ +6.4 (*c* 1, EtOH). ^1^H NMR (600 MHz, DMSO-*d*_6_) δ: 0.67 (s, 3H, CH_3_), 0.71 (d, 3H, *J* = 6.6 Hz, CH_3_), 0.78 (s, 3H, CH_3_), 0.88 (s, 3H, CH_3_), 0.91 (d, 3H, *J* = 6.6 Hz, CH_3_), 0.97 (s, 3H, CH_3_), 1.03 (s, 3H, CH_3_), 1.07 (d, 1H, *J* = 6.6 Hz, CH_3_), 1.67 (s, 3H, CH_3_), 1.17–1.83 (m, 26H, CH, CH_2_), 2.43 (m, 1H, CH), 2.52 (m, 1H, H-19), 2.98 (m, 1H, H-3), 4.02 (d, *J* = 10.8 Hz, 1H, H-28), 4.28 (m, 1H, CHCOOH), 4.53 (d, *J* = 10.8 Hz, 1H, H-28), 4.59 (s, 1H, H-29), 4.73 (s, 1H, H-29), 8.66 (s, 1H, CH-triazol). ^13^C NMR (150 MHz, DMSO-*d*_6_) δ: 15.0, 15.9, 16.1, 16.3, 16.4, 18.4, 19.0, 19.2, 20.2, 20.8, 25.2, 25.3, 27.1, 27.6, 28.6, 29.5, 29.8, 31.8, 34.2, 34.7, 37.2, 37.6, 38.7, 40.9, 42.8, 46.8, 47.6, 48.7, 50.2, 55.31, 55.6, 56.5, 62.5, 72.8, 77.3, 79.7, 110.5, 128.9, 138.4, 150.3, 161.3. IR (KBr, cm^−1^) ν_max_: 1232 (N-N=N), 1456 (N=N), 1541 (C=N), 1717 (C=O), 1734 (C=O), 2870–2968 (C-H), 3385 (O-H). HR-MS (APCI) *m*/*z*: C_38_H_58_N_3_O_5_ [(M − H)^−^], Calc. 636.4455; Found 636.4357.

### 3.4. General Procedure for the Synthesis of Bistriazole Derivatives of Betulin ***6***

A reaction mixture containing 3,28-*O*,*O*’-di(propynoyl)betulin **4** (0.1 g, 0.203 mmol), copper iodide (I) (0.2 eqv, 0.008 g, 0.040 mmol), a corresponding organic azide (2.10 eqv, 0.426 mmol), and dry toluene (4 mL) was stirred under reflux for 72 h. Subsequently, the solution was concentrated under reduced pressure. The crude products were purified by column chromatography using various mixtures of organic solvents to give pure product **6a**–**h**:

*3,28-O,O’-Di[(1-benzyl-1H-1,2,3-triazol-4-yl)carbonyl]betulin* (**6a**). Yield: 48%, m.p. 117–120 °C, ${\left[\alpha \right]}_{D}^{20}$ +2.8 (*c* 1, CHCl_3_). ^1^H NMR (600 MHz, CDCl_3_) δ: 0.77 (s, 3H, CH_3_), 0.79 (s, 3H, CH_3_), 0.87 (s, 3H, CH_3_), 0.91 (s, 3H, CH_3_), 0.98 (s, 3H, CH_3_), 1.67 (s, 3H, CH_3_), 1.10–2.09 (m, 25H, CH, CH_2_), 2.42 (m, 1H, H-19), 4.05 (d, *J* = 10.8 Hz, 1H, H-28), 4.48 (d, *J* = 10.8 Hz, 1H, H-28), 4.53 (s, 1H, H-29), 4.63 (s, 1H, H-29), 4.69 (m, 1H, H-3), 5.60 (s, 4H, CH_2_), 7.22–7.23 (m, 4H, H_Ar_), 7.32–7.33 (m, 6H, H_Ar_), 7.84 (s, 1H, CH-triazol), 7.87 (s, 1H, CH-triazol). ^13^C NMR (150 MHz, CDCl_3_) δ: 14.2, 14.7, 16.0, 16.2, 16.5, 16.7, 18.2, 19.1, 20.8, 21.1, 22.7, 23.8, 25.2, 27.1, 28.0, 29.6, 29.8, 34.1, 37.1, 37.7, 38.2, 38.4, 40.9, 42.7, 46.7, 47.7, 48.9, 50.3, 54.4, 54.5, 55.4, 60.4, 63.6, 81.9, 109.9, 129.9, 126.9, 127.1, 128.2, 129.1, 129.2, 129.3, 133.8, 134.0, 140.6, 150.1, 160.5, 160.6, IR (KBr, cm^−1^) ν_max_: 1261–1144 (N-N=N), 1457 (N=N), 1540 (C=N), 1734 (C=O). HR-MS (APCI) *m*/*z*: C_50_H_64_N_6_O_4_ [(M − H)^−^], Calc. 811.4911; Found 811.4914.

*3,28-O,O’-Di[1-(4-fluorobenzyl-1H-1,2,3-triazol-4-yl)carbonyl]betulin* (**6b**). Yield: 35%, m.p. 155–158 °C, ${\left[\alpha \right]}_{D}^{20}$ +8.8 (*c* 1, CHCl_3_). ^1^H NMR (600 MHz, CDCl_3_) δ: 0.80 (s, 3H, CH_3_), 0.82 (s, 3H, CH_3_), 0.88 (s, 3H, CH_3_), 0.89 (s, 3H, CH_3_), 0.98 (s, 3H, CH_3_), 1.67 (s, 3H, CH_3_), 1.10–2.09 (m, 22H, CH, CH_2_), 2.42 (m, 1H, H-19), 4.05 (d, *J* = 10.8 Hz, 1H, H-28), 4.48 (d, *J* = 10.8 Hz, 1H, H-28), 4.53 (s, 1H, H-29), 4.63 (s, 1H, H-29), 4.69 (m, 1H, H-3), 5.48 (s, 4H, CH_2_), 7.00–7.04 (m, 4H, H_Ar_), 7.22–7.24 (m, 4H, H_Ar_), 7.84 (s, 1H, CH-triazol), 7.88 (s, 1H, CH-triazol). ^13^C NMR (150 MHz, CDCl_3_) δ: 14.8, 16.0, 16.2, 16.8, 18.2, 27.1, 28.1, 34.7, 37.1, 37.7, 38.2, 38.4, 40.9, 42.7, 47.8, 48.9, 50.3, 53.7, 53.8, 63.7, 82.0, 116.3, 116.4, 116.5, 130.1, 130.2. IR (KBr, cm^−1^) ν_max_: 1228–1197 (N-N=N), 1456 (N=N), 1541–1512 (C=N), 1724 (C=O), 2945 (C=C), 1105 (C-F). HR-MS (APCI) *m*/*z*: C_50_H_62_F_2_N_6_O_4_ , [(M − H)^−^], Calc. 847.4722; Found 847.4729 (Figure S7).

*3,28-O,O’-Di[1-(4-cyanobenzyl-1H-1,2,3-triazol-4-yl)carbonyl]betulin* (**6c**). Yield: 35%, m.p. 186–190 °C, ${\left[\alpha \right]}_{D}^{20}$ +16.4 (*c* 1, CHCl_3_). ^1^H NMR (600 MHz, CDCl_3_) δ: 0.90 (s, 3H, CH_3_), 0.92 (s, 3H, CH_3_), 0.98 (s, 3H, CH_3_), 1.00 (s, 3H, CH_3_), 1.16 (s, 3H, CH_3_), 1.72 (s, 3H, CH_3_), 1.10–2.09 (m, 25H, CH, CH_2_), 2.20 (m, 1H, H-19), 4.15 (d, *J* = 10.8 Hz, 1H, H-28), 4.59 (d, *J* = 10.8 Hz, 1H, H-28), 4.63 (s, 1H, H-29), 4.73 (s, 1H, H-29), 4.79 (m, 1H, H-3), 5.68 (s, 4H, CH_2_), 7.39–7.41 (m, 4H, H_Ar_), 7.71–7.73 (m, 4H, H_Ar_), 8.01 (s, 1H, CH-triazol), 8.05 (s, 1H, CH-triazol). ^13^C NMR (150 MHz, CDCl_3_) δ: 16.8, 18.2, 19.1, 20.8, 21.1, 23.8, 25.1, 27.1, 28.1, 29.6, 29.8, 34.1, 34.7, 37.1, 37.7, 38.2, 38.4, 41.0, 42.7, 46.7, 47.8, 48.8, 50.3, 53.7, 55.4, 60.4, 63.8, 82.2, 110.1, 113.2, 113.3, 118.0, 127.2, 127.3, 128.5, 133.1, 140.0, 141.0, 141.4, 150.0, 160.3, 160.9. IR (KBr, cm^−1^) ν_max_: 1228–1192 (N-N=N), 1456 (N=N), 1541 (C=N), 1728 (C=O), 2945 (C=C), 2229 (C=N). HR-MS (APCI) *m*/*z*: C_52_H_62_N_8_O_4_ , [(M − H)^−^], Calc. 861.4816; Found 861.4812.

*3,28-O,O’-Di[(1-phenylthiomethyl-1H-1,2,3-triazol-4-yl)carbonyl]betulin* (**6d**). Yield: 59%, m.p. 127–131 °C, ${\left[\alpha \right]}_{D}^{20}$ +11.2 (*c* 1, CHCl_3_). ^1^H NMR (600 MHz, CDCl_3_) δ: 0.82 (s, 3H, CH_3_), 0.83 (s, 3H, CH_3_), 0.87 (s, 3H, CH_3_), 0.93 (s, 3H, CH_3_), 0.99 (s, 3H, CH_3_), 1.67 (s, 3H, CH_3_), 1.10–2.09 (m, 24H, CH, CH_2_), 2.42 (m, 1H, H-19), 4.04 (d, *J* = 10.8 Hz, 1H, H-28), 4.47 (d, *J* = 10.8 Hz, 1H, H-28), 4.54 (s, 1H, H-29), 4.64 (s, 1H, H-29), 4.68 (m, 1H, H-3), 5.59 (s, 4H, CH_2_), 7.25–7.27 (m, 10H, H_Ar_), 7.89 (s, 1H, CH-triazol), 7.95 (s, 1H, CH-triazol). ^13^C NMR (150 MHz, CDCl_3_) δ: 16.1, 16.2, 16.7, 18.2, 23.8, 25.2, 27.1, 28.1, 29.6, 29.8, 34.1, 34.7, 37.1, 37.7, 38.2, 38.4, 40.9, 42.8, 46.7, 47.8, 48.9, 50.3, 54.3, 55.4, 63.6, 82.1, 110.0, 126.6, 129.1, 129.7, 131.2, 131.3, 132.4, 132.5, 150.0, 160.9. IR (KBr, cm^−1^) ν_max_: 1224–1195 (N-N=N), 1456–1438 (N=N), 1541 (C=N), 1724 (C=O), 2943 (C=C). HR-MS (APCI) *m*/*z*: C_50_H_64_N_6_O_4_S_2_ [(M − H)^−^], Calc. 875.4352, Found 875.4350.

*3,28-O,O’-Di[1-(3′-deoxythymidine-5′-yl-1H-1,2,3-triazol-4-yl)carbonyl]betulin* (**6e**). Yield: 51%, m.p. 224–227 °C, ${\left[\alpha \right]}_{D}^{20}$ +0.9 (*c* 1, CHCl_3_). ^1^H NMR (600 MHz, CDCl_3_) δ: 0.87 (s, 3H, CH_3_), 0.88 (s, 3H, CH_3_), 0.95 (s, 3H, CH_3_), 1.00 (s, 3H, CH_3_), 1.03 (s, 3H, CH_3_), 1.69 (s, 3H, CH_3_), 1.84 (s, 3H, CH_3_-AZT), 1.10–2.09 (m, 25H, CH, CH_2_), 2.20 (m, 1H, H-19), 2.65–2.69 (m, 2H, AZT), 2.76–2.79 (m, 2H, AZT), 3.69–3.72 (m, 2H, AZT), 4.02 (d, *J* = 10,8 Hz, 1H, H-28), 4.28 (t, *J* = 4,8 Hz, 2H, AZT), 4.58 (d, *J* = 10.8 Hz, 1H, H-28), 4.60 (s, 1H, H-29), 4.63 (m, 1H, H-3), 4.75 (s, 1H, H-29), 5.28–5.30 (m, 2H, AZT), 5.45–5.48 (m, 2H, AZT), 6.43–6.46 (m, 2H, AZT), 7.83 (s, 2H, AZT), 8.96 (s, 1H, CH-triazol), 9.02 (s, 1H, CH-triazol), 11.38 (s, 2H, NH-AZT). ^13^C NMR (150 MHz, CDCl_3_) δ: 12.7, 15.0, 16.1, 16.4, 17.0, 19.0, 28.1, 37.1, 37.7, 40.9, 42.8, 46.8, 47.5, 49.9, 60.2, 61.2, 84.3, 84.7, 110.2, 129.3, 136.8, 139.2, 139.5, 150.9, 160.9, 164.2. IR (KBr, cm^−1^) ν_max_: 1273–1199 (N-N=N), 1473 (N=N), 1541–1512 (C=N), 1693 (C=O), 1681 (C=O), 2927 (C-H), 3408–3373 (N-H), 2106 (C-N). HR-MS (APCI) *m*/*z*: C_56_H_76_N_10_O_12_ [(M − H)^−^], Calc. 1079.5566; Found 1079.5564.

*3,28-O,O’-Di[1-(1-deoxy-β-D-glucopyranosyl-1H-1,2,3-triazol-4-yl)carbonyl]betulin* (**6f**). Yield: 95%, oil, ${\left[\alpha \right]}_{D}^{20}$ −0.3 (*c* 1, CHCl_3_). ^1^H NMR (600 MHz, CDCl_3_) δ: 0.87 (s, 6H, 2 × CH_3_), 0.95 (s, 3H, CH_3_), 0.99 (s, 3H, CH_3_), 1.08 (s, 3H, CH_3_), 1.69 (s, 3H, CH_3_), 1.11–1.72 (m, 25H, CH, CH_2_), 1.99 (m, 2H, OH), 2.99 (m, 1H, H-19), 3.09 (m, 2H, OH), 3.19 (m, 2H, OH), 3.26 (m, 1H, H-3), 3.41 (m, 2H, OH), 3.47 (m, 2H, OH), 3.68 (m, 2H, OH), 3.71 (m, 2H, CH-sugar), 3.86 (m, 1H, H-3), 4.02 (d, *J* = 10.8 Hz, 1H, H-28), 4.47 (m, 1H, CH-sugar), 4.56 (d, *J* = 10.8 Hz, 1H, H-28), 4.58 (s, 1H, H-29), 4.65 (m, 2H, CH-sugar), 4.74 (s, 1H, H-29), 5.21 (m, 2H, CH-sugar), 5.35 (m, 2H, CH-sugar), 5.45 (m, 2H, CH-sugar), 5.61 (m, 2H, CH-sugar), 9.08 (s, 1H, CH-triazol), 9.08 (s, 1H, CH-triazol). ^13^C NMR (150 MHz, DMSO-*d*_6_) δ: 15.0, 16.1, 16.4, 17.2, 18.2, 19.3, 23.9, 25.2, 25.7, 27.1, 28.1, 29.4, 29.6, 37.1, 37.6, 38.2, 40.8, 42.6, 42.8, 46.8, 47.5, 48.7, 49.9, 55.0, 61.2, 62.6, 69.9, 70.0, 72.4, 73.8, 77.0, 77.2, 79.6, 80.6, 81.4, 88.3, 90.6, 110.4, 129.9, 129.1, 139.2, 139.4, 150.3, 160.4, 160.9. IR (KBr, cm^−1^) ν_max_: 1236 (N-N=N), 1458 (N=N), 1543 (C=N), 1732 (C=O), 2941 (C=C), 3406 (OH). HR-MS (APCI) *m*/*z*: C_48_H_72_N_6_O_14_ [(M − H)^−^], Calc. 955.5028, Found 955.5024.

*3,28-O,O’-Di-[(1-ethylacetyl-1H-1,2,3-triazol-4-yl)carbonyl]betulin* (**6g**). Yield 71%, m.p. 133–136 °C, ${\left[\alpha \right]}_{D}^{20}$ −7.6 (*c* 1, CHCl_3_). ^1^H NMR (600 MHz, CDCl_3_) δ: 0.83 (s, 3H, CH_3_), 0.85 (s, 3H, CH_3_), 0.90 (s, 3H, CH_3_), 0.94 (s, 3H, CH_3_), 1.02 (s, 3H, CH_3_), 1.24 (m, 6H, 2 × CH_3_), 1.64 (s, 3H, CH_3_), 1.10–1.98 (m, 25H, CH, CH_2_), 2.45 (m, 1H, H-19), 4.08 (d, *J* = 10.8 Hz, 1H, H-28), 4.22 (m, 4H, 2 × OCH_2_), 4.52 (d, *J* = 10.8 Hz, 1H, H-28), 4.54 (s, 1H, H-29), 4.65 (s, 1H, H-29), 4.73 (m, 1H, H-3), 5.15 (s, 4H, 2 × CH_2_), 8.11 (s, 1H, CH-triazol), 8.15 (s, 1H, CH-triazol). ^13^C NMR (150 MHz, CDCl_3_) δ: 14.1, 14.2, 14.8, 16.1, 16.2, 16.8, 18.2, 19.1, 20.8, 23.8, 25.2, 27.1, 28.1, 29.6, 29.8, 34.1, 34.7, 37.1, 37.7, 38.2, 38.4, 40.9, 42.8, 46.7, 47.8, 48.9, 50.3, 51.0, 55.4, 62.8, 62.81, 63.7, 82.1, 110.0, 128.5, 128.7, 140.7, 141.0, 150.1, 160.3, 160.9, 165.7, 165.7. IR (KBr, cm^−1^) ν_max_: 1207 (N-N=N), 1467 (N=N), 1543 (C=N), 1751 (C=O), 2872–2947 (C-H). HR-MS (APCI) *m*/*z*: C_44_H_64_N_6_O_8_ [(M − H)^−^], Calc. 803.4707; Found 803.4704.

*3,28-O,O’-Di[1-(3-hydroxypropyl-1H-1,2,3-triazol-4-yl)carbonyl]betulin* (**6h**). Yield: 78%, m.p. 172–176 °C, ${\left[\alpha \right]}_{D}^{20}$ −1.5 (*c* 1, EtOH). ^1^H NMR (600 MHz, CDCl_3_) δ: 0.83 (s, 3H, CH_3_), 0.85 (s, 3H, CH_3_), 0.91 (s, 3H, CH_3_), 0.93 (s, 3H, CH_3_), 1.00 (s, 3H, CH_3_), 1.18 (t, 4H, *J* = 7.2 Hz, 2 × CH_2_OH), 1.69 (s, 3H, CH_3_), 1.25–2.12 (m, 25H, CH, CH_2_), 2.44 (m, 1H, H-19), 3.60 (m, 4H, 2 × CH_2_), 3.65 (t, 4H, *J* = 7.2 Hz, 2 × CH_2_), 4.08 (d, *J* = 10.8 Hz, 1H, H-28), 4.49 (br.s, 2H, 2 × OH), 4.51 (d, *J* = 10.8 Hz, 1H, H-28), 4.54 (s, 1H, H-29), 4.65 (s, 1H, H-29), 4.71 (m, 1H, H-3), 7.99 (s, 1H, CH-triazol), 8.03 (s, 1H, CH-triazol). ^13^C NMR (150 MHz, CDCl_3_) δ: 14.8, 16.1, 16.2, 16.8, 18.2, 18.5, 19.1, 20.8, 23.8, 25.2, 25.3, 27.1, 28.1, 29.6, 29.9, 32.3, 32.4, 34.1, 34.7, 37.1, 37.7, 38.1, 38.4, 40.9, 42.8, 46.7, 47.2, 47.3, 47.8, 48.9, 50.3, 55.4, 58.6, 63.6, 82.0, 110.0, 127.7, 150.1, 160.3, 161.2. IR (KBr, cm^−1^) ν_max_: 1220–1203 (N-N=N), 1456 (N=N), 1541 (C=N), 1724 (C=O), 2872–2947 (C-H), 3435 (O-H). HR-MS (APCI) *m*/*z*: C_42_H_64_N_6_O_6_ [(M − H)^−^], Calc. 747.4809; Found 747.4802 (Figure S8).

### 3.5. Biological Activities

#### 3.5.1. Cells and Viruses

The synthesized compounds, dissolved in DMSO (20 mg/mL), were tested against VSV—vesicular stomatitis virus (strain Indiana; ATTC VR-1238™, *Rhabdoviridae*); HSV-1—herpes simplex virus type-1 (HSV-1-strain MacIntyre; ATTC VR-539™, *Herpesviridae*); ECBO—cytopathogenic bovine orphan virus (BEV-1, ECBO–Enteric, strain LCR-4; ATTC VR-248™, *Picornaviridae*); HAdV-5—human adenovirus type-5 (strain Adenoid 75; ATTC VR-5™, *Adenoviridae*) in A549 (ATCC^®^ CCL-185™)—human lung adenocarcinoma epithelial cell line culture

#### 3.5.2. Cytotoxicity Assay: Determination of Cytotoxic Concentration CC_50_

The cytotoxic effect of compounds on A549 cells was determined by the MTT method, as previously described [36]. The MTT assay is the enzyme-based method, which use the 3-(4,5-dimethylthiazol-2-yl)-2,5-diphenyltetrazolium bromide as a reductive coloring agent. Briefly, subconfluent monolayers of A549 cells (2 × 10^4^ cell/well in Dulbecco’s Minimal Essential Medium (DMEM) supplemented with 5% fetal bovine serum (FBS), antibiotics (100 U/mL penicillin and 100 μg/mL streptomycin), and 2 mM L-glutamine (all from Sigma Aldrich, St. Louis, MO, USA) were incubated in 96-multi-well plates in the presence of dilutions of the compounds ranging from 100 μg/mL to 0.5 μg/mL, in triplicate for 48 h at 37 °C in a humidified atmosphere with 5% CO_2_. After 48 h, 20 μL of MTT solution (Promega, Madison, WI, USA) was added to each well, and the microplates were kept at 37 °C/5% CO_2_ for 3 h. Then, the solubilization/stop solution containing SDS-HCl (100 μL/well) was added, and the absorbance values of the wells were measured at 570 nm using a 96-well plate reader (Multiskan RC spectrophotometer, Thermo Labsystems, Waltham, MA, USA). Cell viability (%) was calculated for each concentration as Abs treated/Abs control × 100, where Abs treated and Abs control are the absorbance readings for the wells with and without the tested compounds, respectively. The 50% cytotoxic concentration (CC_50_) was defined as the concentration that reduced cell viability by 50% with respect to controls without drugs. The CC_50_ value was calculated from the corresponding dose–response curves.

#### 3.5.3. Virucidal Activity

The virucidal activity was measured in vitro incubating various strains of viruses with the compounds. Briefly, the viruses (VSV−1 × 10^7^ TCID_50_/mL, HSV−1 × 10^6^ TCID_50_/mL, ECBO−1 × 10^5^ TCID_50_/mL, HAdV−5 1 × 10^5^ TCID_50_/mL) were incubated for 2 h at room temperature with a non-toxic (CC_20_) concentration of each compound. Simultaneously, the same amount of virus was incubated with the culture medium as a control. After incubation, viruses were titrated (10^−1^ to 10^−8^) and plated on A549 cell monolayers seeded in 96-well plates 24 h before the experiment (2 × 10^4^ cell/well). The plates were incubated for 48 h (for VSV and HSV-1) and 72 h (for ECBO and HAdV-5) at 37 °C in a humidified atmosphere with 5% CO_2_. The cytopathic effect (CPE) was observed under microscope. Scoring of CPE from 0 (no CPE) to 4 (100% cell destruction) was performed.

#### 3.5.4. Pretreatment Assays

To assess the effect of the pretreatment with the compounds, A549 cell monolayers seeded in 96-well plates were treated for 24 h at 37 °C, with the CC_20_ of each compound in triplicate. Then, the medium was removed, and the cell monolayers were infected with 100 TCID_50_/well of each virus. After 30 min at 37 °C for VSV and HSV-1, and 1 h for ECBO and HAdV-5, the viral inoculum was removed. Plates were washed and finally overlaid with 2% DMEM (100 μL per well), and then incubated 48 h for VSV and HSV-1, and 72 h for ECBO and HAdV-5. Finally, the cytopathic effect (CPE) was observed under microscope and determined by the MTT method.

#### 3.5.5. Time of Addition Assay

To study the effect of the compounds in the adsorption and post-adsorption events, two different treatments with the compounds were carried out. First, when the derivatives were present: (i) only during the adsorption period (Adsorption); (ii) after adsorption and until the end of the experiment (Post-Adsorption). Briefly, A549 cell monolayers cultured in 96-well plates were then infected with 100 TCID_50_/well of each virus in the presence or absence of each compound, and further incubated at 37 °C (for the designated time for each virus), allowing only the adsorption step of the viral particles to the cells (Adsorption). Cell monolayers were then washed and incubated with 2% DMEM. In the Post-Adsorption experiments, A549 cell monolayers were infected with 100 TCID_50_/well of each virus, and after adsorption, cells were washed, and derivatives were added to the wells. CPE was assessed 48 h und 72 h under microscope and by the MTT method. All of the experiments mentioned above were performed in parallel with a drug effective against the used virus (Acyclovir, Cidofovir, Ribavirin, Interferon-γ) and betulin **1** to test the suitability of the assay. Each assay was run in triplicate.

#### 3.5.6. Measurement of Inhibition Activity on Tumor Cell Proliferation

Antiproliferative activities of the compounds were measured by the MTT assay using A549 cells and a human ovarian cancer cell line SKOV-3 (adenocarcinoma ATCC^®^ HTB77™). A total of 2 × 10^3^ cells in DMEM medium supplemented with 5% FBS were placed in each well of a 96-well flat-bottom plate. After 24 h of incubation at 37 °C in 5% CO_2_, the growth medium was replaced by media containing different concentrations of the derivatives. Control cultures received cisplatin 0.1–10 μg/mL, and blank wells contained 100 μL of growth medium with no cells. After 96 h of incubation, cell proliferation was determined by the colorimetric MTT assay (570 nm) reading for each concentration compared with the control. At least three replicates for each sample were used to determine the cell proliferation.

#### 3.5.7. Anticancer Activity

The cytotoxic activity of the compounds was measured in vitro using following human cancer cell lines: human ductal carcinoma T47D (ATCC, Rockville, MD, USA), human adenocarcinoma MCF-7, glioblastoma SNB-19 (DSMZ, Braunschweig, Germany), human malignant melanoma Colo-829 (ATCC, Rockville, MD, USA), and human amelanotic melanoma C-32 (ATCC, Rockville, MD, USA). The cells were grown in DMEM (Lonza, Basel, Switzerland) growth medium containing 10% fetal bovine serum (FBS) (Biological Industries, Cromwell, CT, USA), penicillin (10.000 U/mL) and streptomycin (10 mg/mL) (Lonza, Basel, Switzerland). Cells were seeded (5 × 10^3^ cells/well) into 96-well plates (Nunc Thermo Fisher Scientific, Waltham, MA, USA) and incubated for 24 h at 37 °C in a 5% CO_2_ atmosphere at 95% humidity. The culture medium was replaced with a fresh aliquot containing the tested compounds (0.01–100 μg/mL). Next, the cells were incubated with the compounds for another 72 h. The cytotoxic activity was evaluated using a WST-1 test (Roche Diagnostics GmbH, Mannheim, Germany), which is based on the cleavage of the bright red-colored stable tetrazolium salt WST-1 to dark red soluble formazan by mitochondrial dehydrogenases in viable cells. The amount of formazan correlates directly with the amount of metabolically active cells in the culture, and is measured by absorbance (λ = 450 nm).

#### 3.5.8. Antimicrobial Activity

The antibacterial and antifungal activities of compounds were evaluated using the following strains: *Staphylococcus aureus* (ATCC 25923), *Enterococcus faecalis* (ATCC 29212), *Escherichia coli* (ATTC 25922), *Pseudomonas aeruginosa* (ATTC 27853), *Klebsiella pneumoniae* ATTC 700603), and *Candida albicans* (ATTC 10231). Cells were cultured for 24–48 h on Columbia agar or Sabouraud agar (*C. albicans*) in a saline solution (0.9% NaCl) and adjusted to a 0.5 McFarland density standard (1.5 × 10^8^ CFU/mL). The final density of microorganisms was approximately 3 × 10^5^ CFU/mL. The solutions of compounds were added to the medium at various concentrations (0.06 μM–2 mM). The MIC (minimal inhibitory concentration) was defined as the lowest concentration of the examined compounds inhibiting the growth of the cell strains after 18 h incubation at 35 °C in ambient air. The experiments were performed in triplicate.

## 4. Conclusions

In conclusion, novel mono- and bis-1,4-disubstituted triazole hybrids of betulin were synthesized by the Huisgen cycloaddition reaction. The acetylenic and triazole compounds were tested for their antiviral activity using a vesicular stomatitis virus (VSV), herpes simplex virus type-1 (HSV-1), bovine picornavirus (ECBO) and human adenovirus type-5 (hAdV-5). The transformation of acetylenic moiety to triazole ring was necessary to demonstrate antiviral activity. Several of the hybrid conjugates showed a promising antiviral activity against ECBO strain LCR-4 in A549 cells. The most active compound was **6h**, which contained a 3-hydroxypropyl substituted 1,2,3-triazole ring. The cytotoxic results showed that the bistriazole **6b** had potential activity against the human ductal carcinoma T47D as compared with cisplatin. In the case of the bistriazoles 6e and 6f, both which contain substituents associated with the occurrence of antiviral activity, the total decay of cytotoxic effect against all of the tested cancer cell lines was observed. Only, the triazole **5e** containing a 3′-deoxythymidine-5′-yl moiety showed antibacterial activity against *Klebsiella pneumoniae* and *Escherichia coli* strains.

## Figures and Tables

**Table 1:** The selected chemical shifts ^1^H NMR and ^13^C NMR and correlations proton–carbon (HSQC and HMBC experiments) for compound **5a**.

^1^H NMR δ (ppm)	^13^C NMR δ (ppm)	HSQC	HMBC
H-33 7.88	C-33 127.16	7.88–127.16	7.88–140.61/54.49
H-34 5.51	C-34 54.49	5.51–54.49	5.51–133.77

**Table 2:** Antiviral activity of betulin derivatives against the cytopathogenic bovine orphan virus (ECBO) strain LCR-4 in adenocarcinoma epithelial (A549) cells.

Compound	CC_50_ (μg/mL)	EC_50_ (μg/mL)	SI
**5e**	10.3	1.0	10.3
**5f**	100.0	5.0	20.0
**5h**	27.7	0.5	55.5
**5j**	100.0	5.0	20.0
**6f**	100.0	5.0	20.0
**6h**	29.8	0.25	0.25
**Betulin 1**	18.5	0.5	37.0
**Ribavirin**	100.0	10.0	10.0

CC_50_—50% cytotoxic concentration; concentration required to reduce A549 cells viability by 50%. EC_50_—50% effective concentration; concentration required to inhibit ECBO cytopathic effect in A549 cells by 50%. SI—selectivity index, or ratio of CC_50_ to EC_50_.

**Table 3:** Antiproliferative activity of triazoles in the SKOV-3 human ovarian cancer line in comparison to betulin **1** and cisplatin.

Compound	Concentration (μg/mL)	Max. Inhibition (% of Control)
**5a**	25	40
**5b**	10	40
**5d**	10	40
**5e**	50	75
**5f**	25	35
**5j**	10	40
**6h**	50	70
**Betulin 1**	2.5	30
**Ribavirin**	1.5	30

**Table 4:** The cytotoxic activity of derivatives **3**–**4**, **5a**–**k**, **6a**–**h** and cisplatin against tested cell lines: T47D (human ductal carcinoma), MCF-7 (human adenocarcinoma), SNB-19 (glioblastoma), Colo-829 (human malignant melanoma), and C-32 (human amelanotic melanoma).

Compound	Human Cell Line /IC_50_ ± SD [μM]				
	T47D	MCF-7	SNB-19	Colo-829	C-32
**Betulin 1**	Neg	Neg	17.7 ± 1.2	15.3 ± 2.2	Neg
**3**	110.8 ± 0.9	102.1 ± 1.4	4.2 ± 0.3	1.7 ± 0.08	16.7 ± 0.8
**4**	Neg	Neg	13.1 ± 1.3	9.7 ± 1.5	121.2 ± 4.1
**5a**	10.5 ± 0.09	58.8 ± 1.5	96.2 ± 1.2	78.5 ± 8.3	Neg
**5b**	8.5 ± 0.02	Neg	66.1 ± 0.7	129.2 ± 7.7	Neg
**5c**	1.4 ± 0.01	Neg	0.70 ± 0.08	9.7 ± 0.1	Neg
**5d**	52.5 ± 1.4	Neg	13.0 ± 0.09	100.0 ± 5.5	Neg
**5e**	Neg	Neg	Neg	35.1 ± 6.2	75.2 ± 3.7
**5f**	Neg	Neg	Neg	109.5 ± 7.2	Neg
**5g**	135.2 ± 2.6	Neg	92.3 ± 0.9	11.4 ± 0.2	Neg
**5h**	1.3 ± 0.05	15.5 ± 1.1	9.9 ± 0.7	6.2 ± 0.6	80.3 ± 5.8
**5i**	3.63 ± 0.3	6.94 ± 0.8	0.9 ± 0.03	0.6 ± 0.13	7.9 ± 0.9
**5j**	Neg	Neg	Neg	3.3 ± 1.1	Neg
**5k**	7.2 ± 0.2	37.2 ± 0.7	6.3 ± 0.9	8.7 ± 0.5	46.9 ± 2.5
**6a**	Neg	Neg	24.4 ± 1.3	80.7 ± 1.8	Neg
**6b**	0.05 ± 0.01	0.09 ± 0.01	0.08 ± 0.01	Neg	Neg
**6c**	1.1 ± 0.01	60.7 ± 0.6	0.06 ± 0.01	Neg	Neg
**6d**	0.9 ± 0.05	0.9 ± 0.08	0.5 ± 0.02	102.6 ± 4.7	100.4 ± 2.8
**6e**	Neg	Neg	Neg	Neg	Neg
**6f**	Neg	Neg	Neg	Neg	Neg
**6g**	Neg	Neg	Neg	97.6±3.3	Neg
**6h**	62.4 ± 1.1	11.7 ± 1.2	68.3 ± 0.9	6.5 ± 1.0	9.1 ± 0.2
**Cisplatin**	24.9 ± 1.1	5.5 ± 1.0	2.3 ± 0.05	16.8 ± 1.7	12.3 ± 2.1

Neg—negative in the concentration used.

**Table 5:** The minimal inhibitory concentration (MIC) and minimal bactericidal concentration (MBC) of compound **5e** against *Escherichia coli* and *Klebsiella pneumoniae*.

Strains	MIC (μM)	MBC (μM)
*Escherichia coli* ATCC 25922	1.95	7.8
*Klebsiella pneumoniae* ATCC 700603	0.95	3.9

## Supplementary Materials

Supplementary materials can be accessed online.
