# Supplementary material for: Novel Triazole Hybrids of Betulin: Synthesis and Biological Activity Profile
Source: Molecules. 2017 Nov 1;22(11):1876. doi: 10.3390/molecules22111876 (PMC6150379; doi:10.3390/molecules22111876)
Supplement: Supplementary file 1 [file molecules-22-01876-s001.pdf]

Supplementary material

Novel triazole hybrids of betulin: synthesis and biological activity profile

Ewa Bębenek, Maria Jastrzębska, Monika Kadela-Tomanek, Elwira Chrobak, Beata Orzechowska, Katarzyna Zwolińska, Małgorzata Latocha, Anna Mertas, Zenon Czuba, Stanisław Boryczka

Table of Contents

Figure S1: (a) HSQC spectrum, (b) HMBC spectrum of 28-O-(1-Benzyl-1H-[1,2,3]-triazol-4-yl)carbonylbetulin (**5a**)

Figure S2: (a) ^{1}H NMR spectrum, (b) ^{13}C NMR spectrum, (c) IR spectrum of 28-O-(1-Benzyl-1H-[1,2,3]-triazol-4-yl)carbonylbetulin (**5a**)

Figure S3: (a) ^{1}H NMR spectrum, (b) ^{13}C NMR spectrum, (c) IR spectrum of 28-O-{1-(4-Fluorobenzyl)-1H-[1,2,3]-triazol-4-yl}carbonylbetulin (**5b**)

Figure S4: (a) ^{1}H NMR spectrum, (b) ^{13}C NMR spectrum, (c) IR spectrum of 28-O-{1-(4-Cyanobenzyl)-1H-[1,2,3]-triazol-4-yl}carbonylbetulin (**5c**)

Figure S5: (a) ^{1}H NMR spectrum, (b) ^{13}C NMR spectrum, (c) IR spectrum of 28-O-(1-Phenylthiomethyl-1H-[1,2,3]-triazol-4-yl)carbonylbetulin (**5d**)

Figure S6: (a) ^{1}H NMR spectrum, (b) ^{13}C NMR spectrum, (c) IR spectrum of 28-O-{1-(3′-Deoxythymidine-5′-yl)-1H-[1,2,3]-triazol-4-yl}carbonylbetulin (**5e**)

Figure S7: (a) ^{1}H NMR spectrum, (b) ^{13}C NMR spectrum, (c) IR spectrum of 3,28-O,O′-Di{1-(4-fluorobenzyl-1H-[1,2,3]-triazol-4-yl)carbonyl}betulin (**6b**)

Figure S8: (a) ^{1}H NMR spectrum, (b) ^{13}C NMR spectrum, (c) IR spectrum of 3,28-O,O′-Di{1-(3-hydroxypropyl-1H-[1,2,3]-triazol-4-yl)carbonyl}betulin (**6h**)

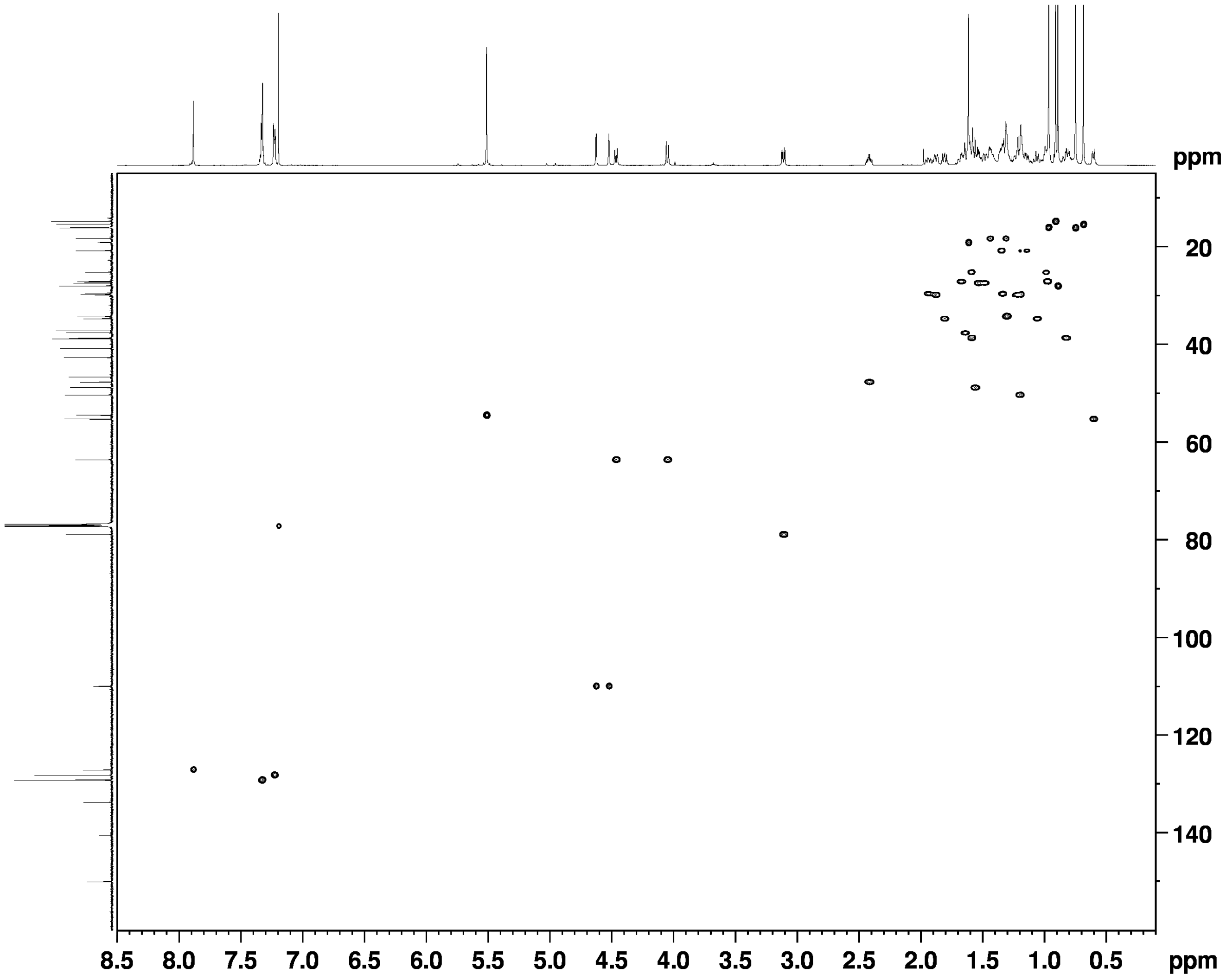

Figure S1 (a): HSQC spectrum of 28-O-(1-Benzyl-1H-[1,2,3]-triazol-4-yl)carbonylbetulin (**5a**)

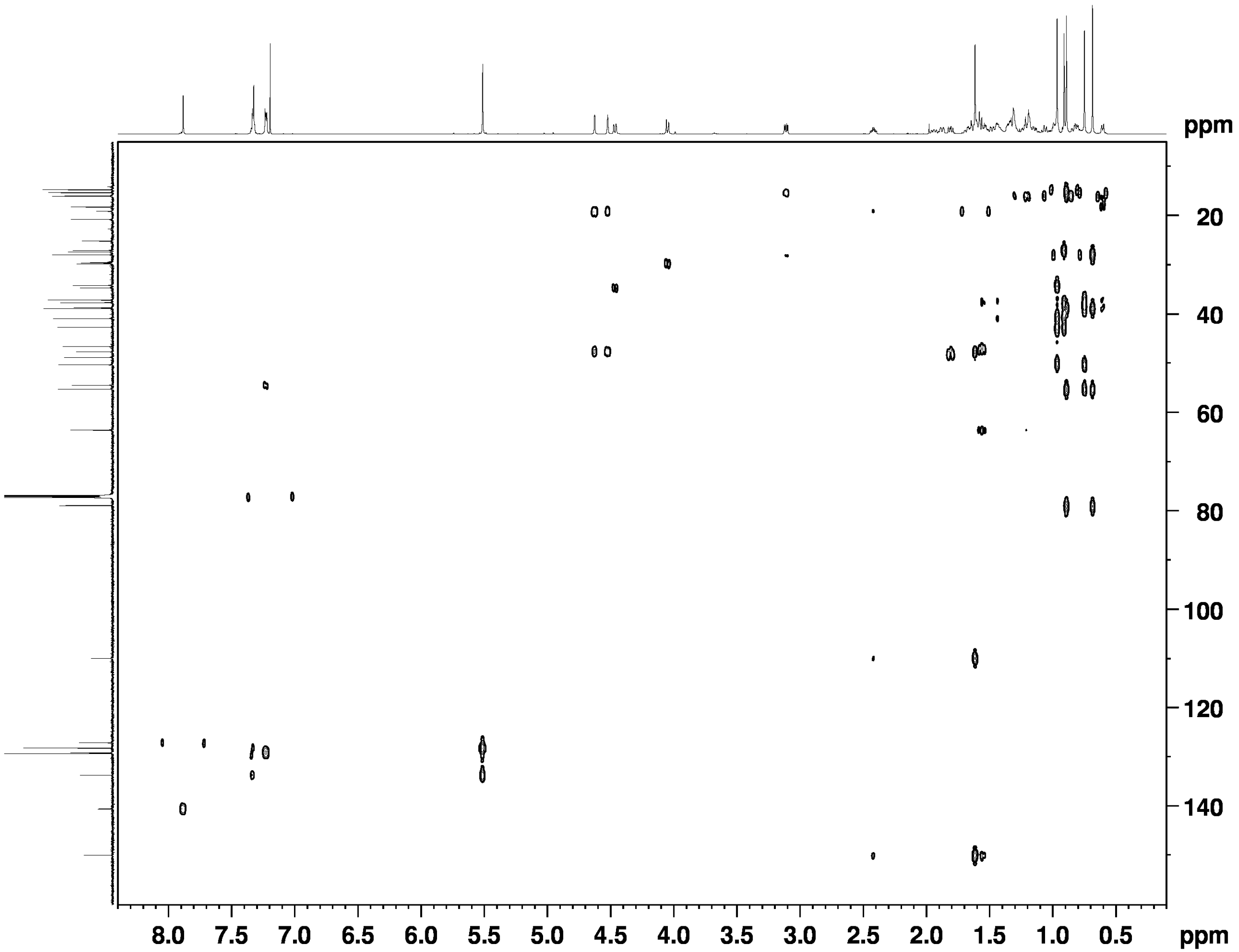

Figure S1 (b): HMBC spectrum of 28-O-(1-Benzyl-1H-[1,2,3]-triazol-4-yl)carbonylbetulin (**5a**)

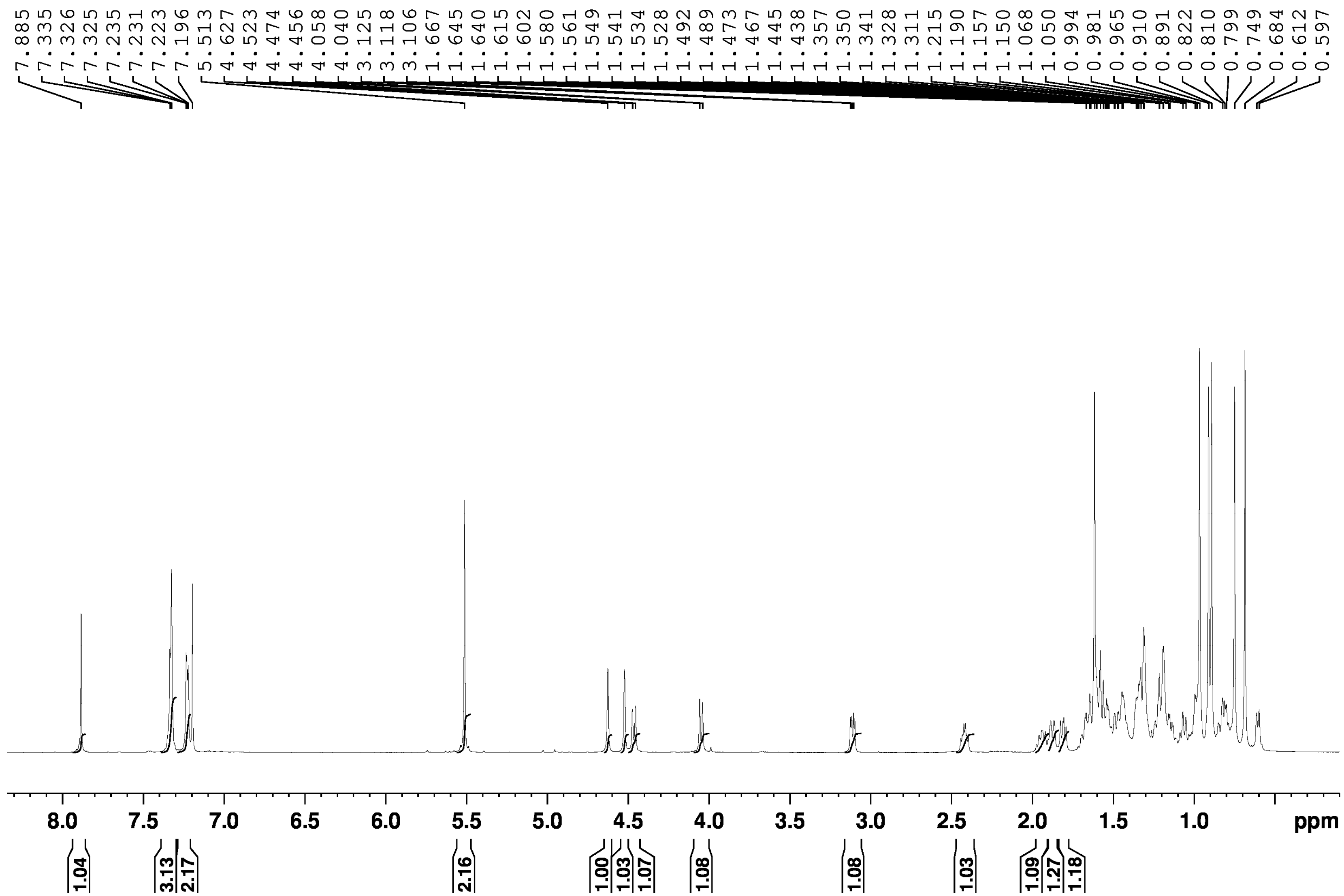

Figure S2 (a): ^{1}H NMR spectrum of 28-O-(1-Benzyl-1H-[1,2,3]-triazol-4-yl)carbonylbetulin (**5a**)

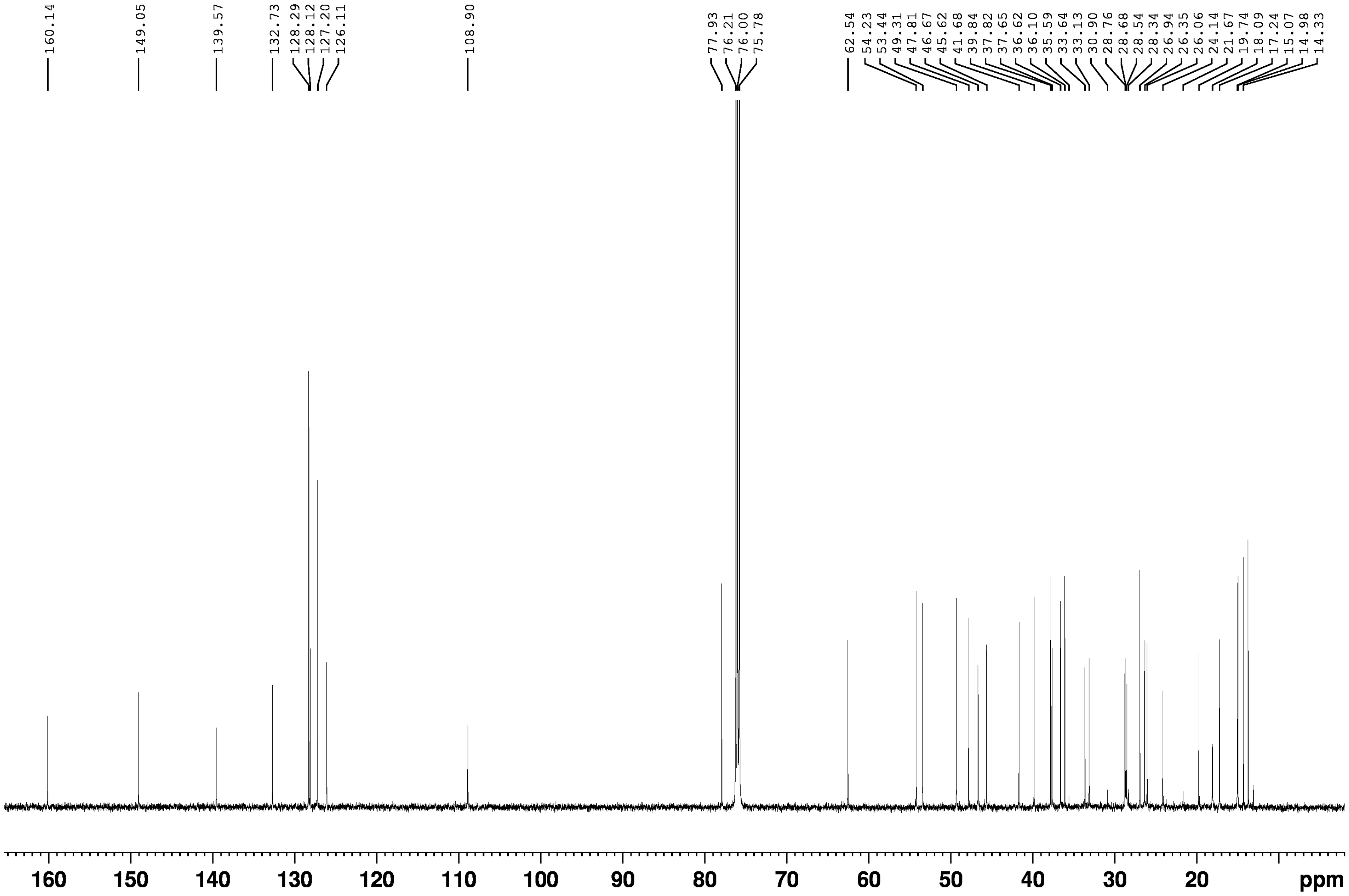

Figure S2 (b): ^{13}C NMR spectrum of 28-O-(1-Benzyl-1H-[1,2,3]-triazol-4-yl)carbonylbetulin (**5a**)

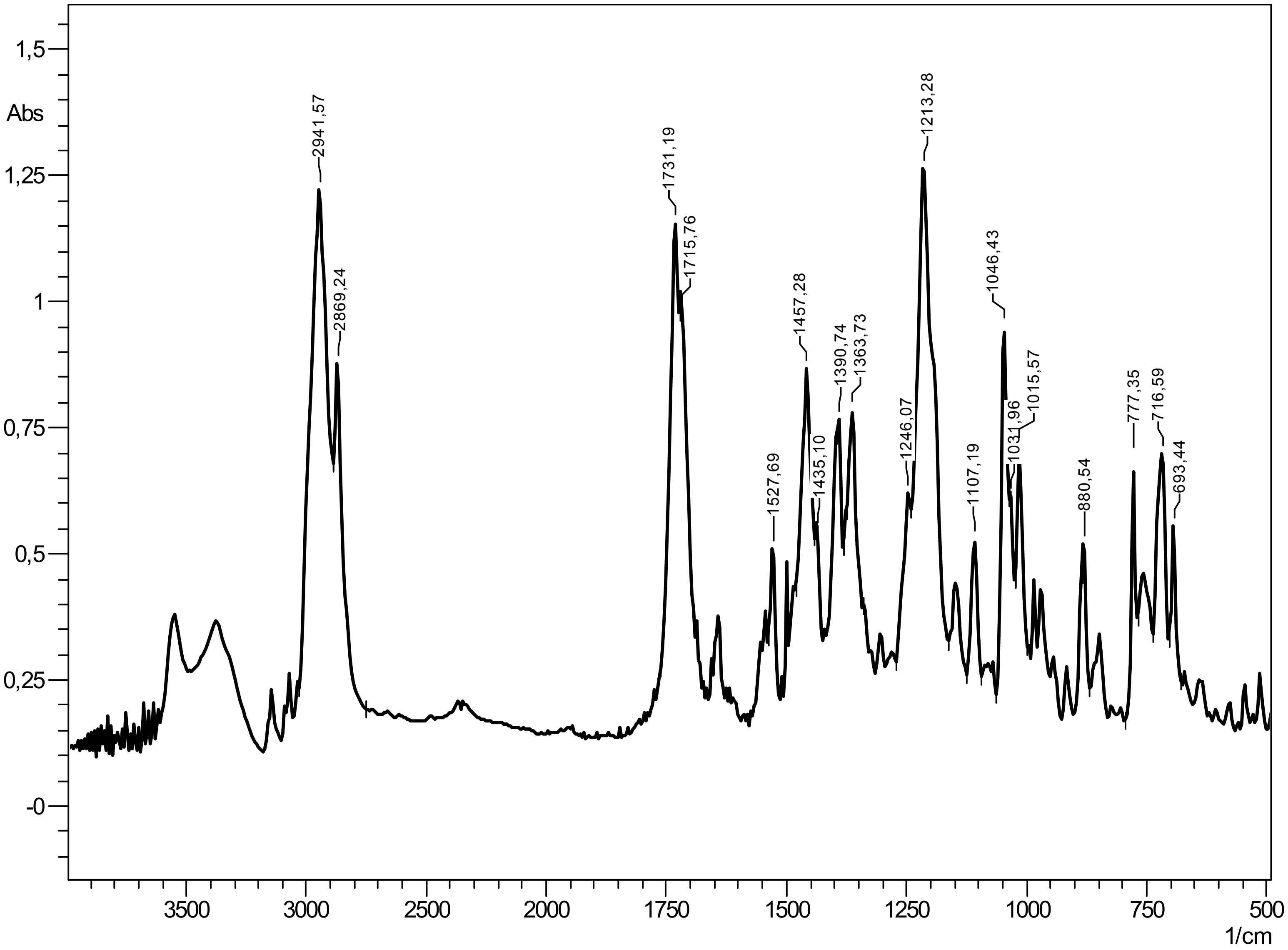

Figure S2 (c): IR spectrum of 28-O-(1-Benzyl-1H-[1,2,3]-triazol-4-yl)carbonylbetulin (**5a**)

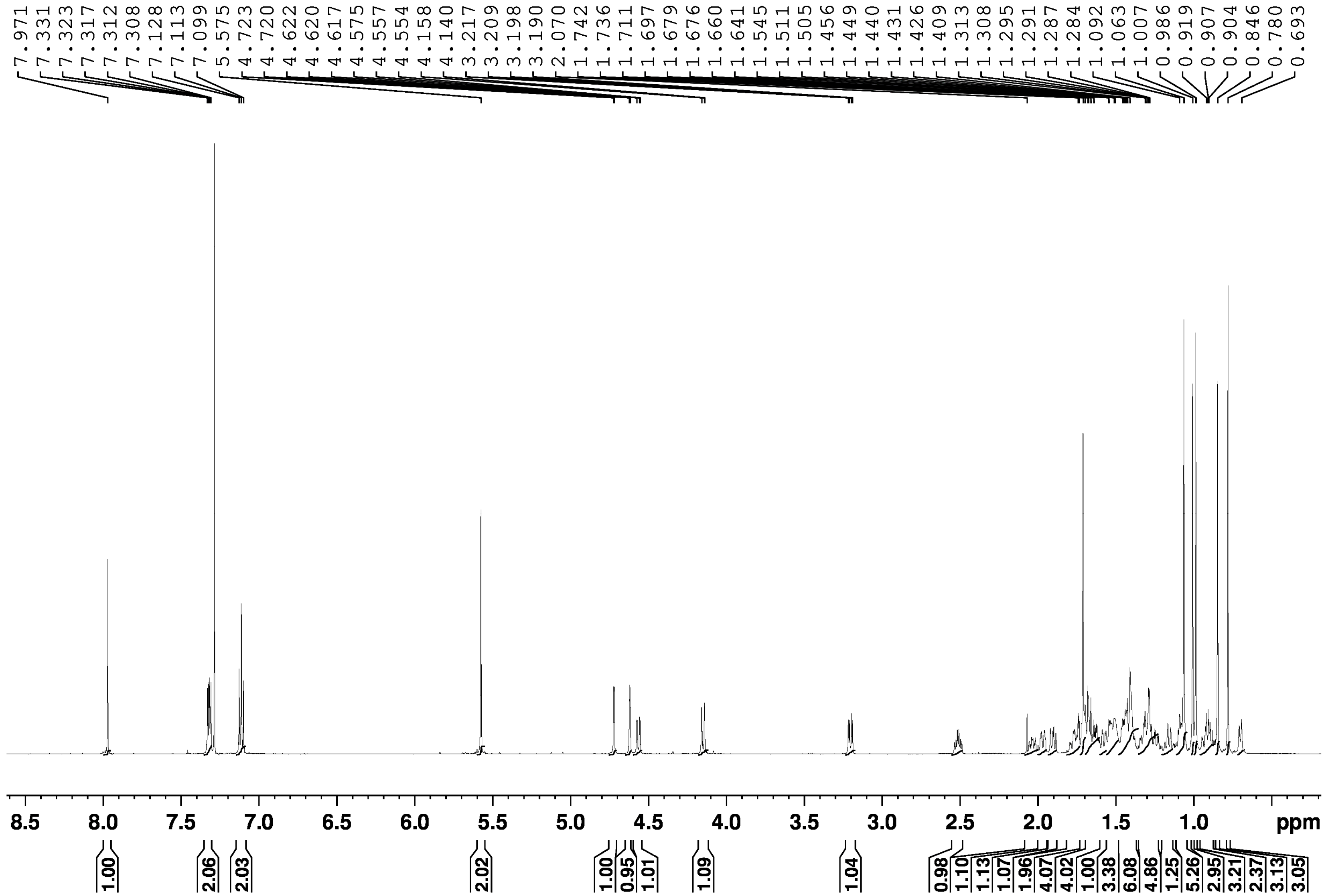

Figure S3 (a): ^{1}H NMR spectrum of 28-O-{1-(4-Fluorobenzyl)-1H-[1,2,3]-triazol-4-yl}carbonylbetulin (**5b**)

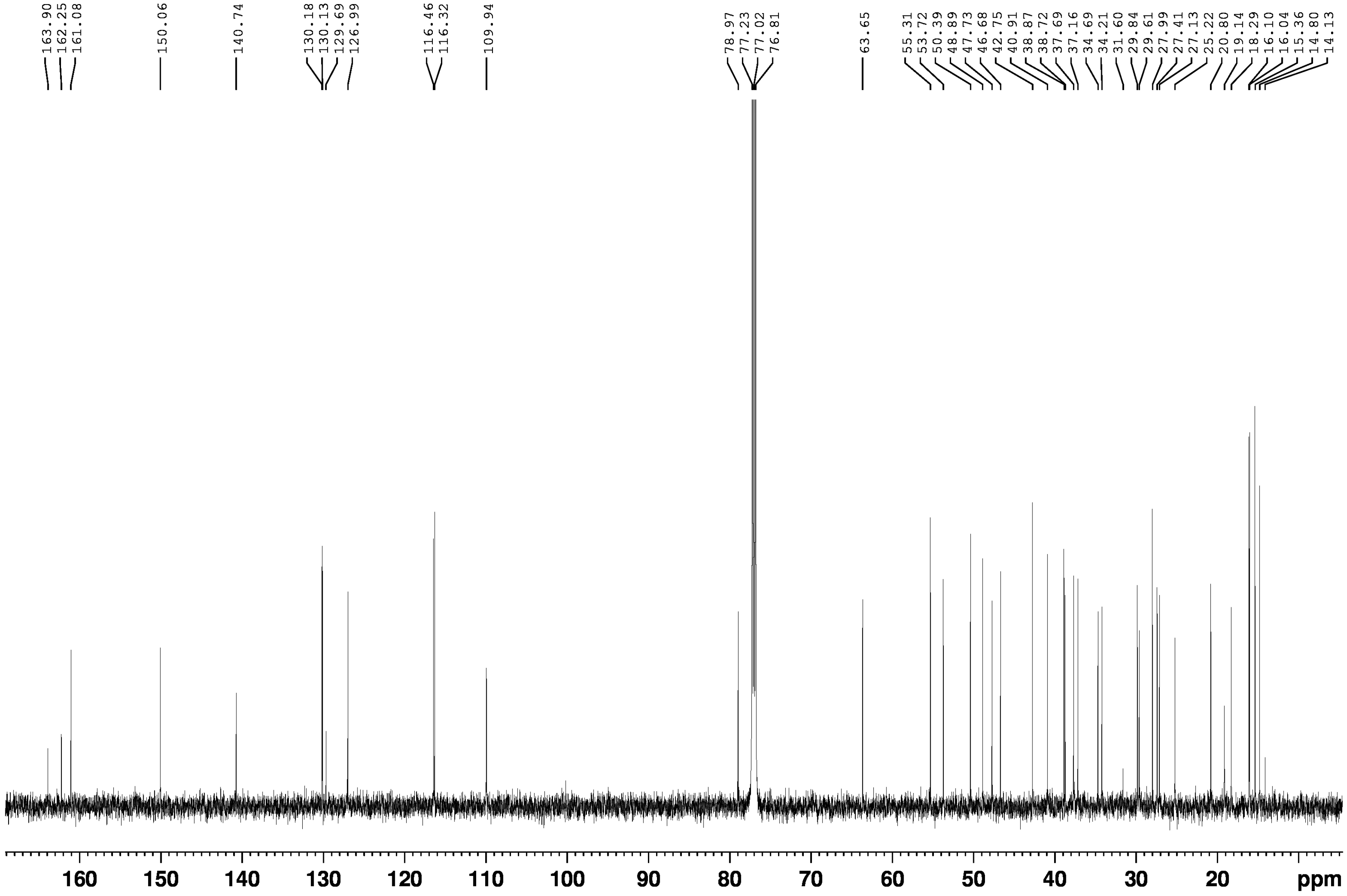

Figure S3 (b): ^{13}C NMR spectrum of 28-O-{1-(4-Fluorobenzyl)-1H-[1,2,3]-triazol-4-yl}carbonylbetulin (**5b**)

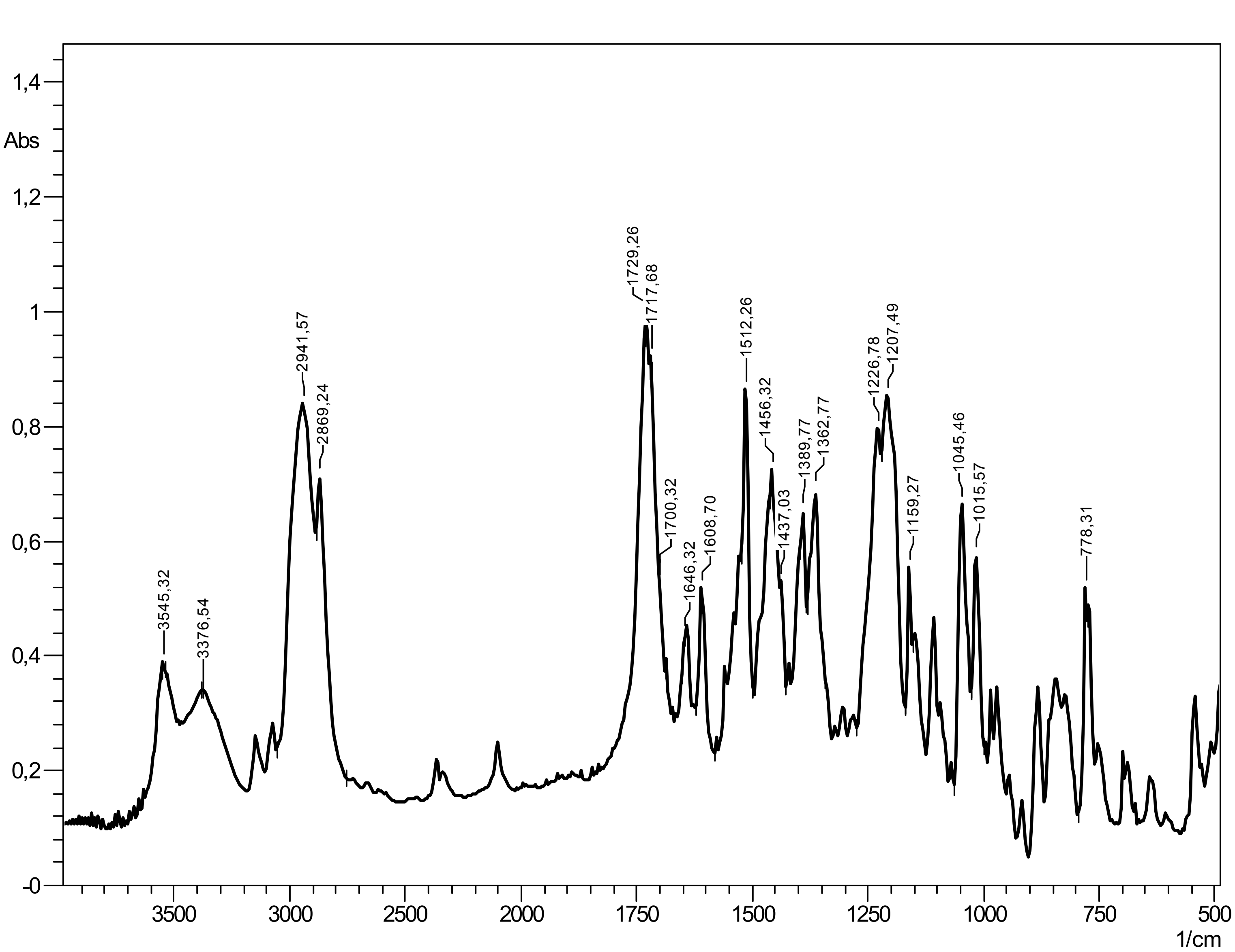

Figure S3 (c): IR spectrum of 28-O-{1-(4-Fluorobenzyl)-1H-[1,2,3]-triazol-4-yl}carbonylbetulin (**5b**)

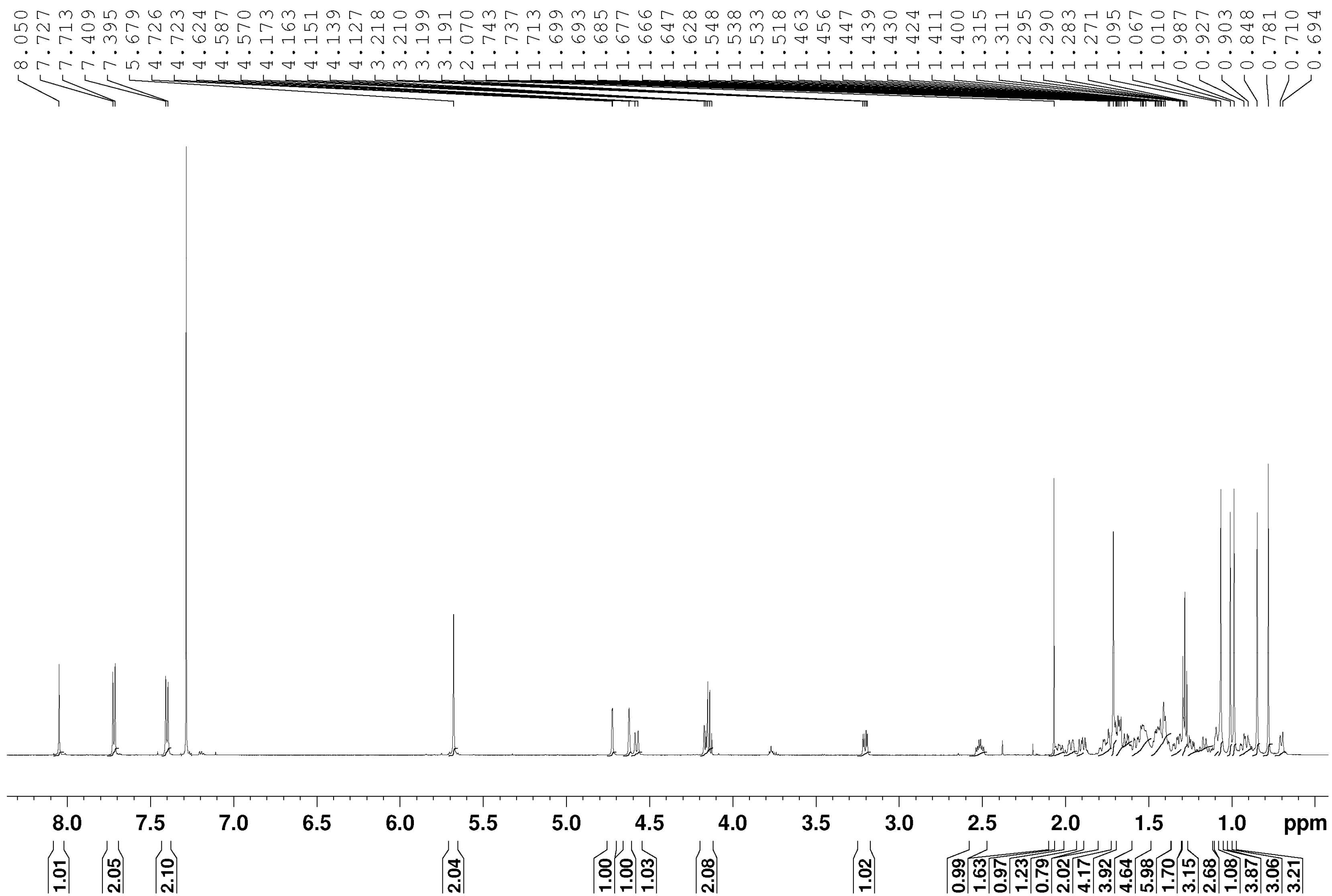

Figure S4 (a): ^{1}H NMR spectrum of 28-O-{1-(4-Cyanobenzyl)-1H-[1,2,3]-triazol-4-yl}carbonylbetulin (**5c**)

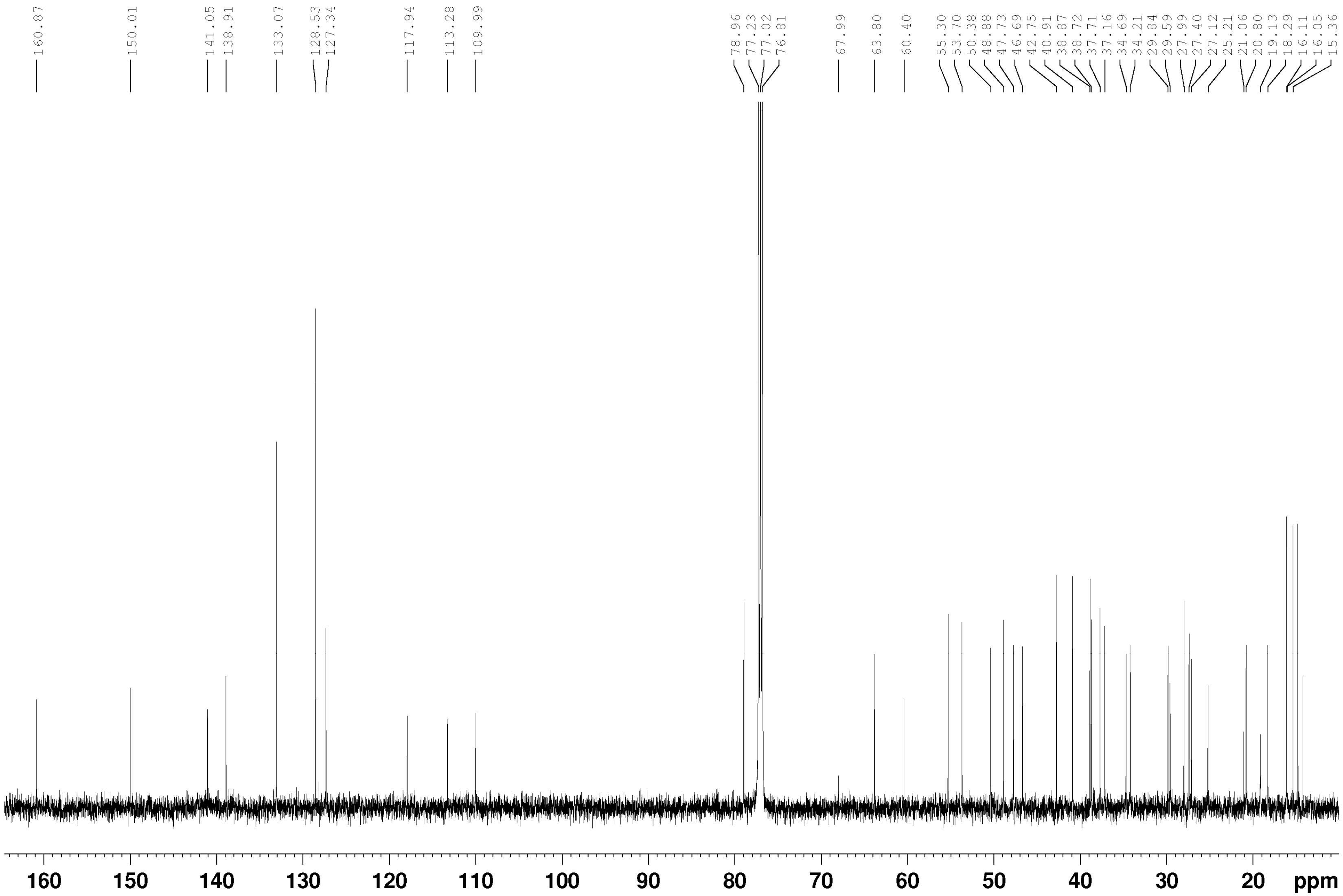

Figure S4 (b): ^{13}C NMR spectrum of 28-O-{1-(4-Cyanobenzyl)-1H-[1,2,3]-triazol-4-yl}carbonylbetulin (**5c**)

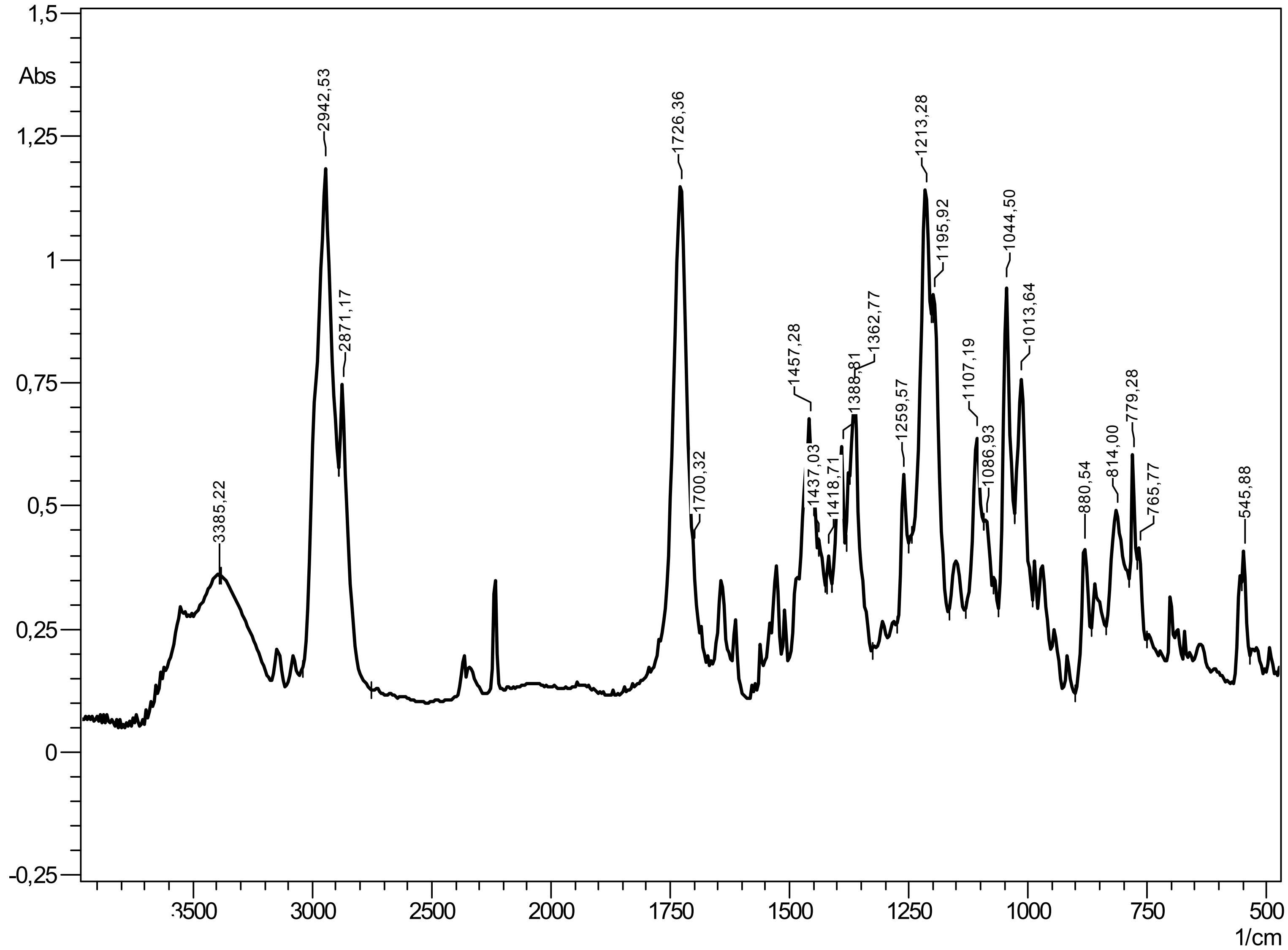

Figure S4 (c): IR spectrum of 28-O-{1-(4-Cyanobenzyl)-1H-[1,2,3]-triazol-4-yl}carbonylbetulin (**5c**)

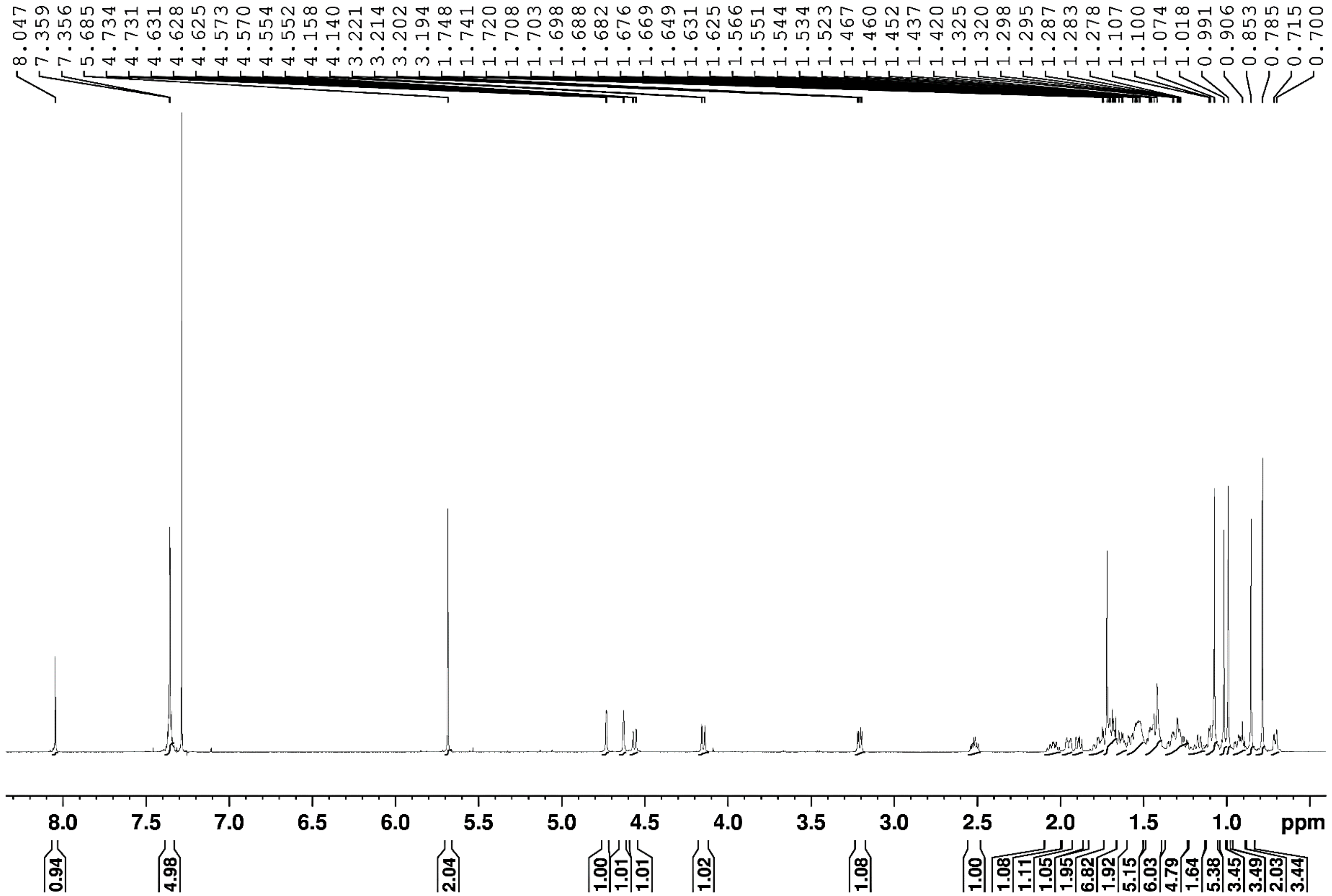

Figure S5 (a): ^{1}H NMR spectrum of 28-O-(1-Phenylthiomethyl-1H-[1,2,3]-triazol-4-yl)carbonylbetulin (**5d)**

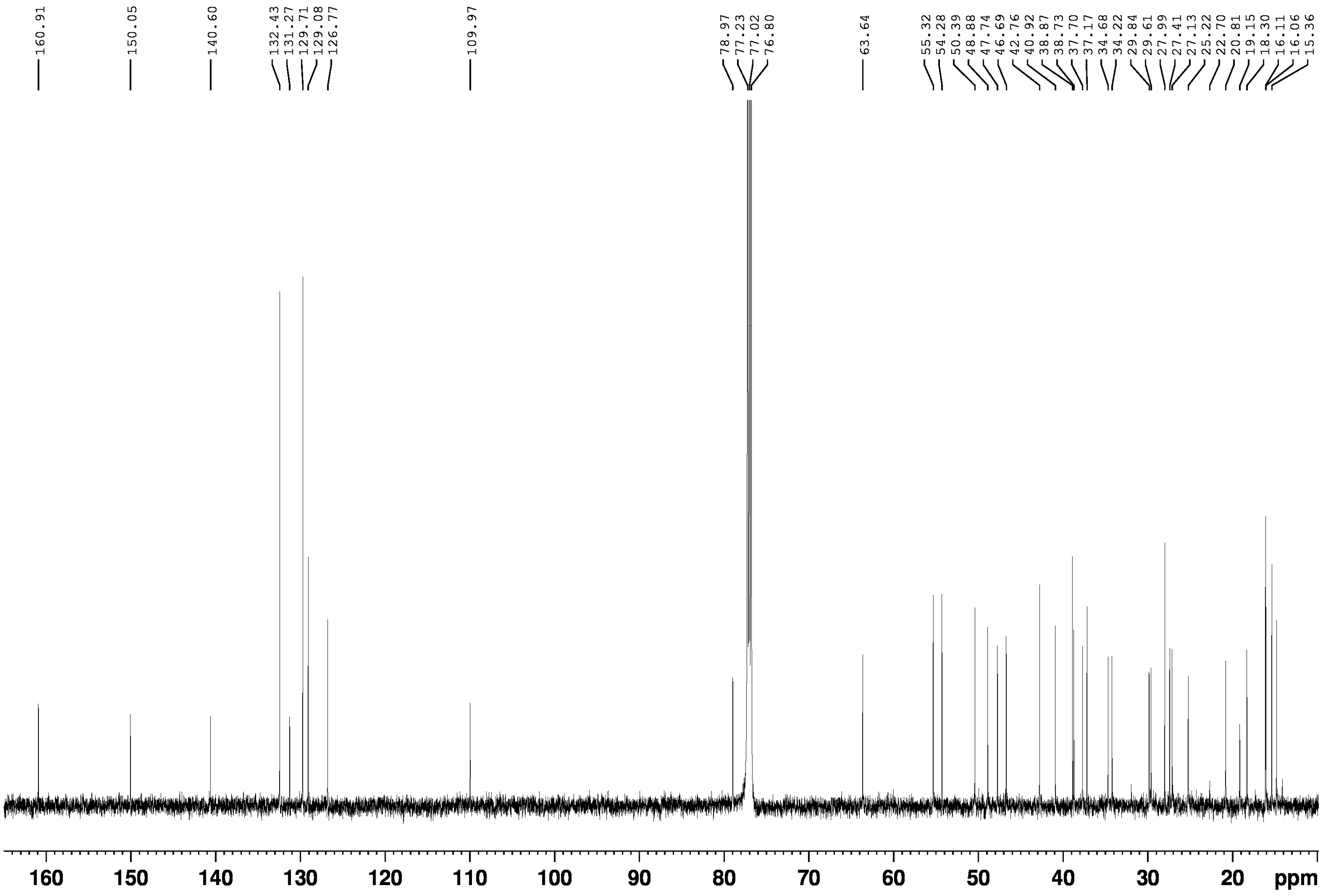

Figure S5 (b): ^{13}C NMR spectrum of 28-O-(1-Phenylthiomethyl-1H-[1,2,3]-triazol-4-yl)carbonylbetulin (**5d**)

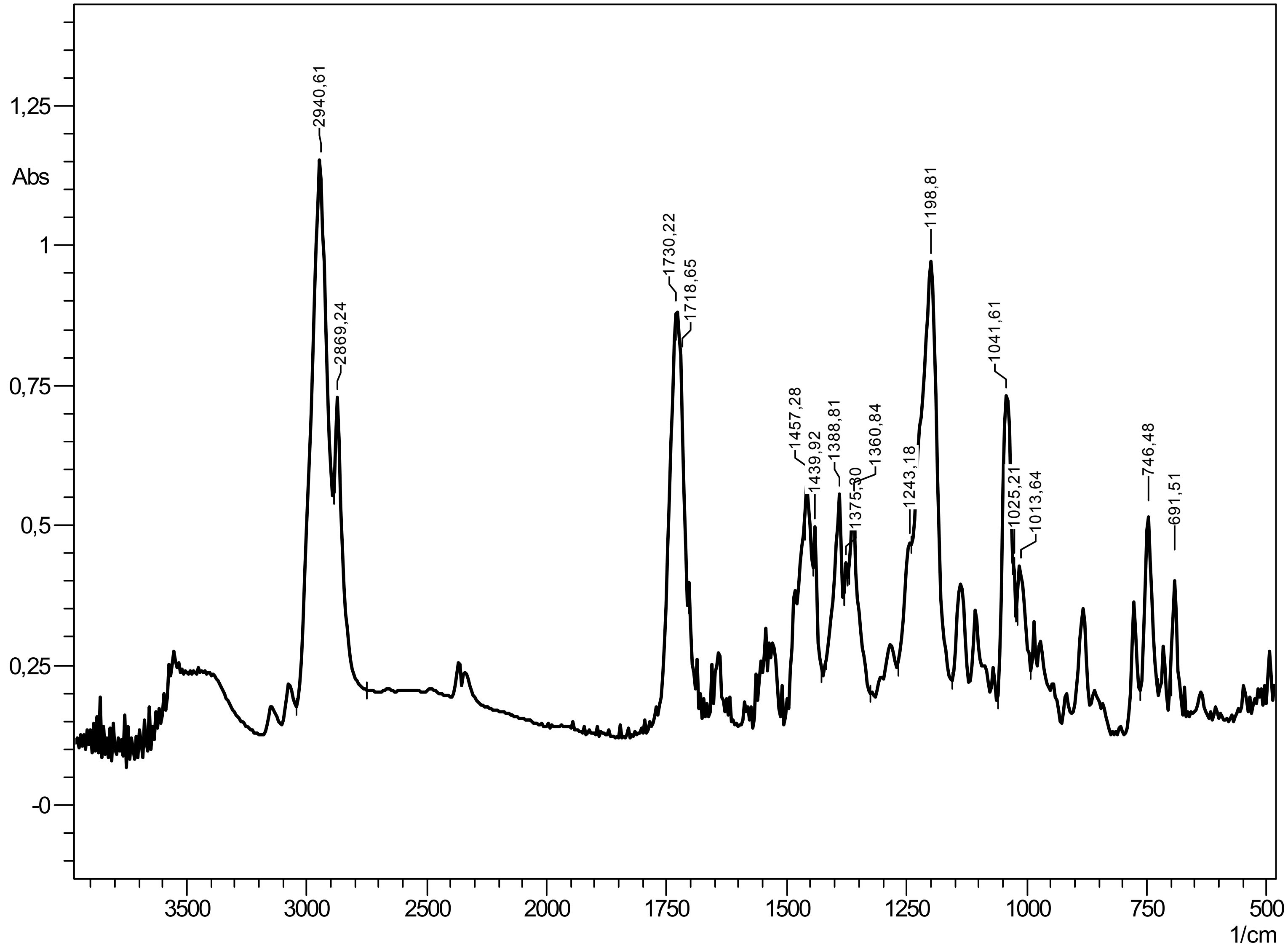

Figure S5 (c): IR spectrum of 28-O-(1-Phenylthiomethyl-1H-[1,2,3]-triazol-4-yl)carbonylbetulin (**5d**)

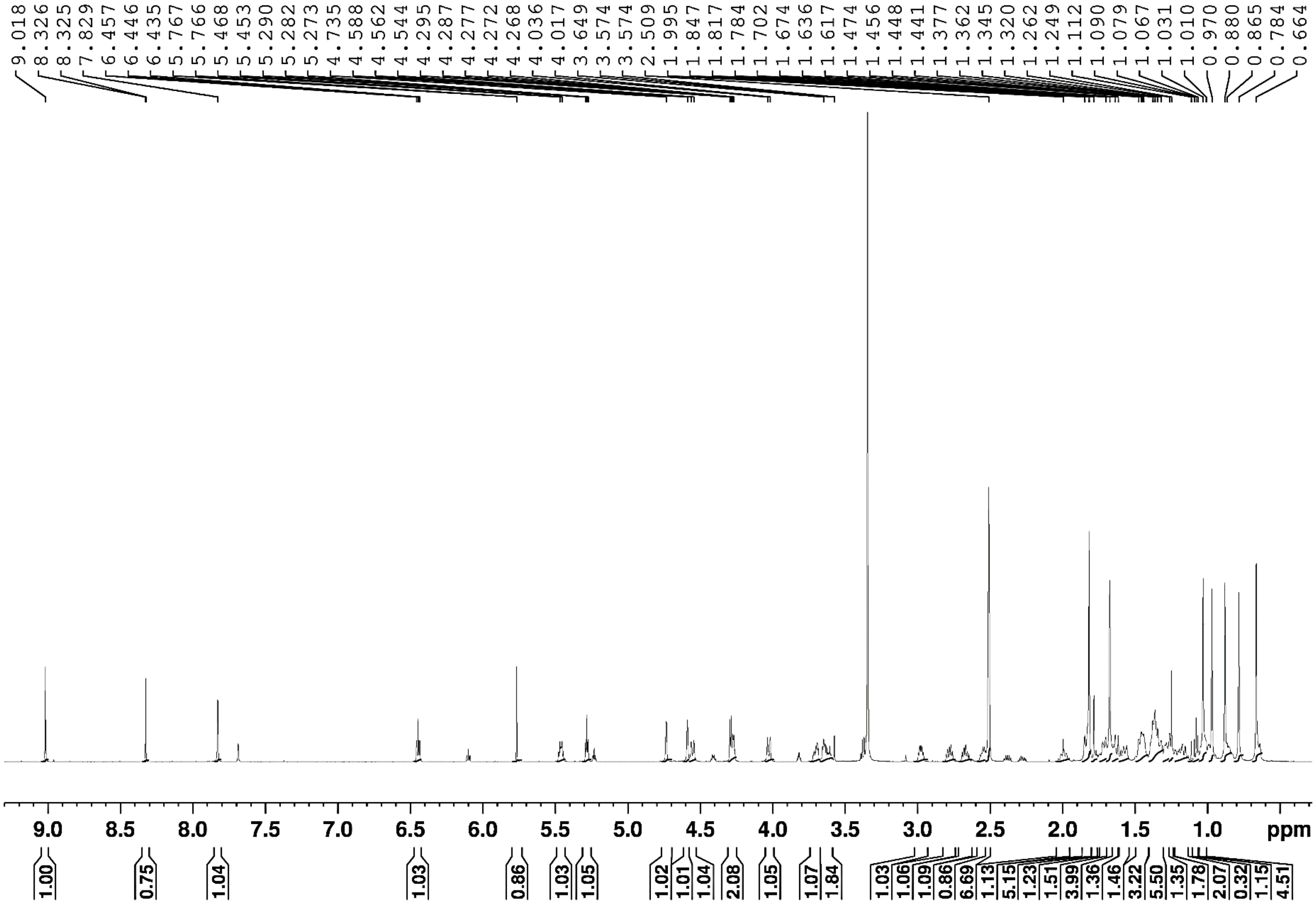

Figure S6 (a): ^{1}H NMR spectrum of 28-O-{1-(3′-Deoxythymidine-5′-yl)-1H-[1,2,3]-triazol-4-yl}carbonylbetulin (**5e**)

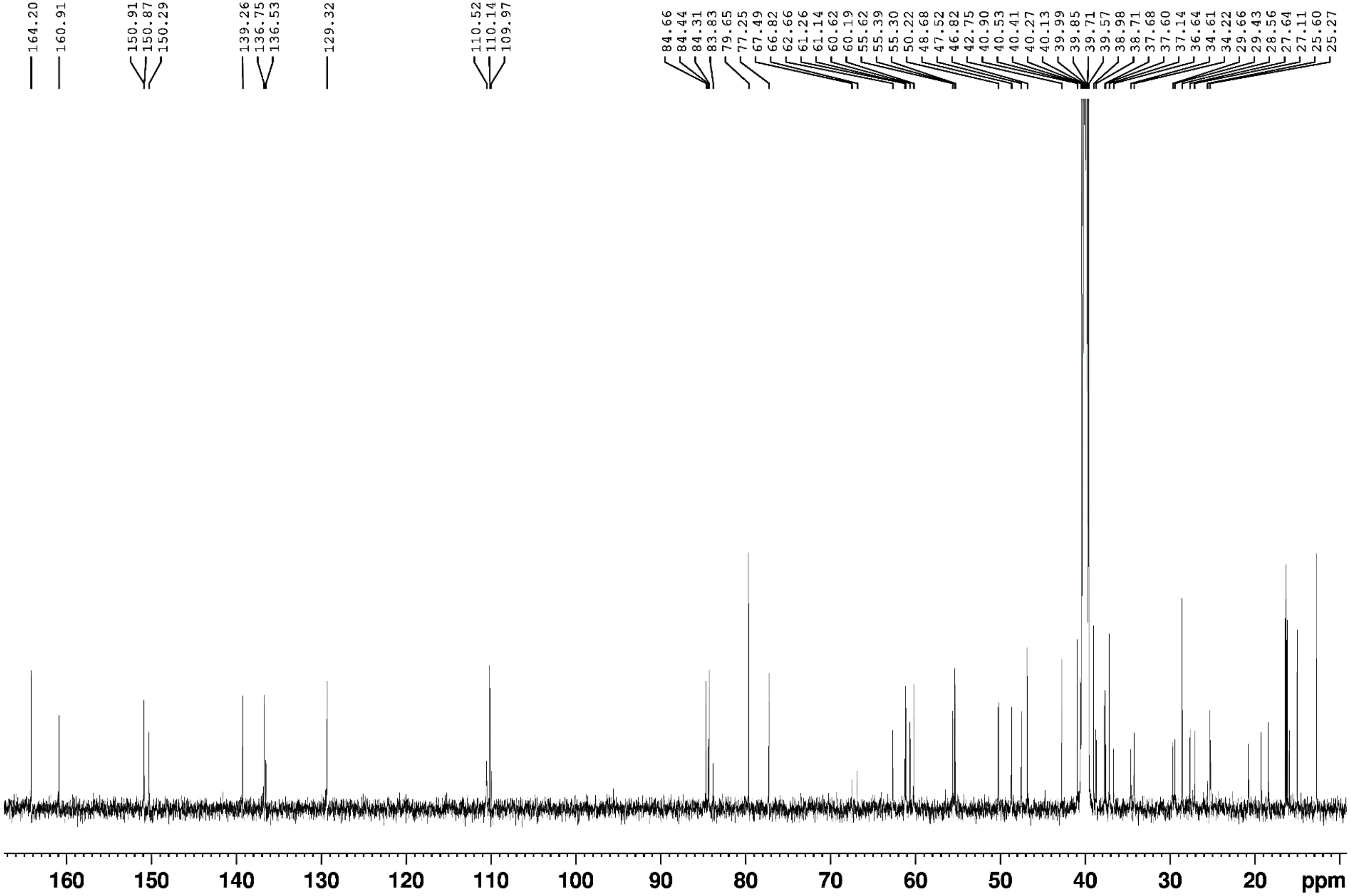

Figure S6 (b): ^{13}C NMR spectrum of 28-O-{1-(3′-Deoxythymidine-5′-yl)-1H-[1,2,3]-triazol-4-yl}carbonylbetulin (**5e**)

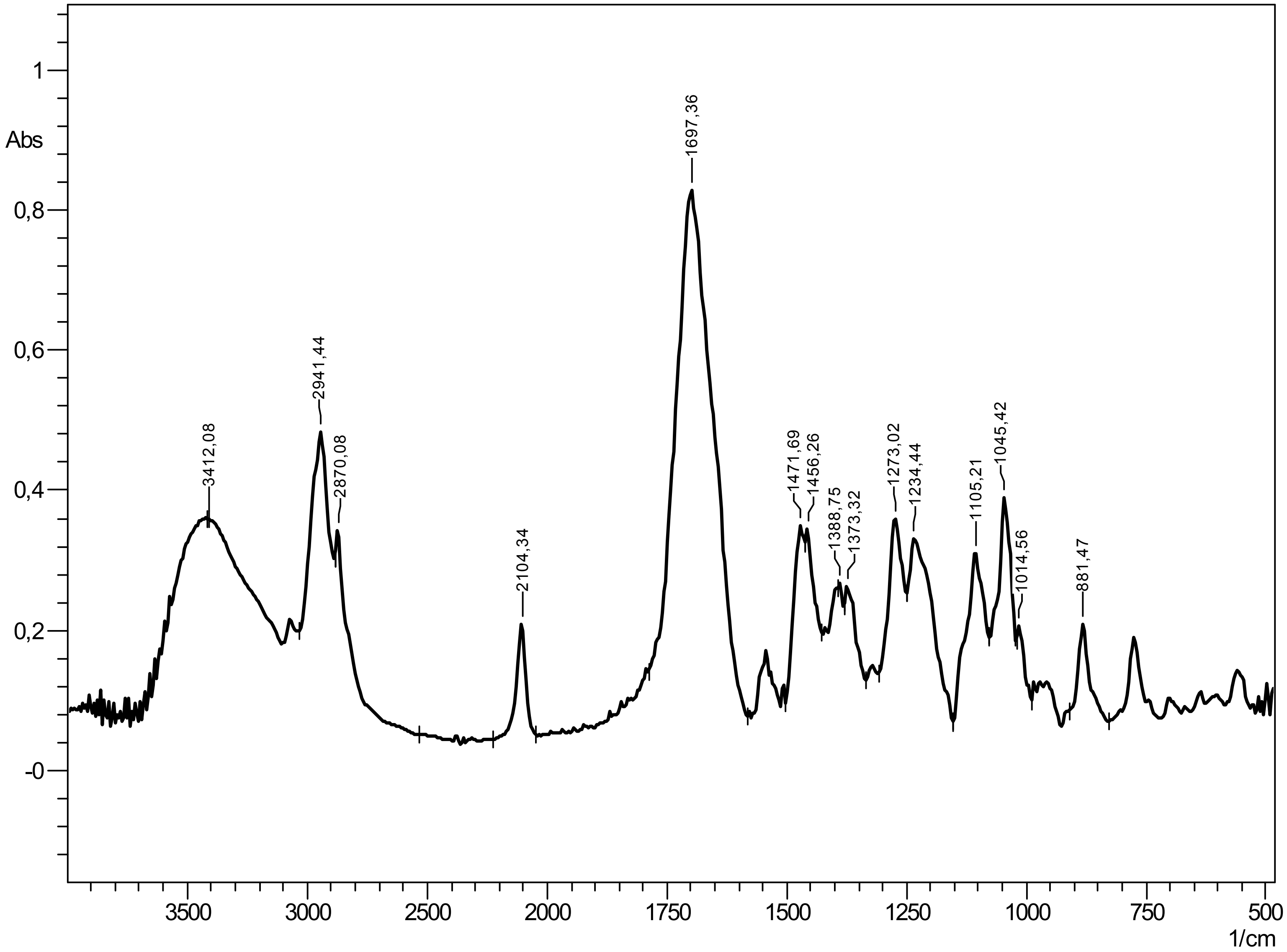

Figure S6 (c): IR spectrum of 28-O-{1-(3′-Deoxythymidine-5′-yl)-1H-[1,2,3]-triazol-4-yl}carbonylbetulin (**5e**)

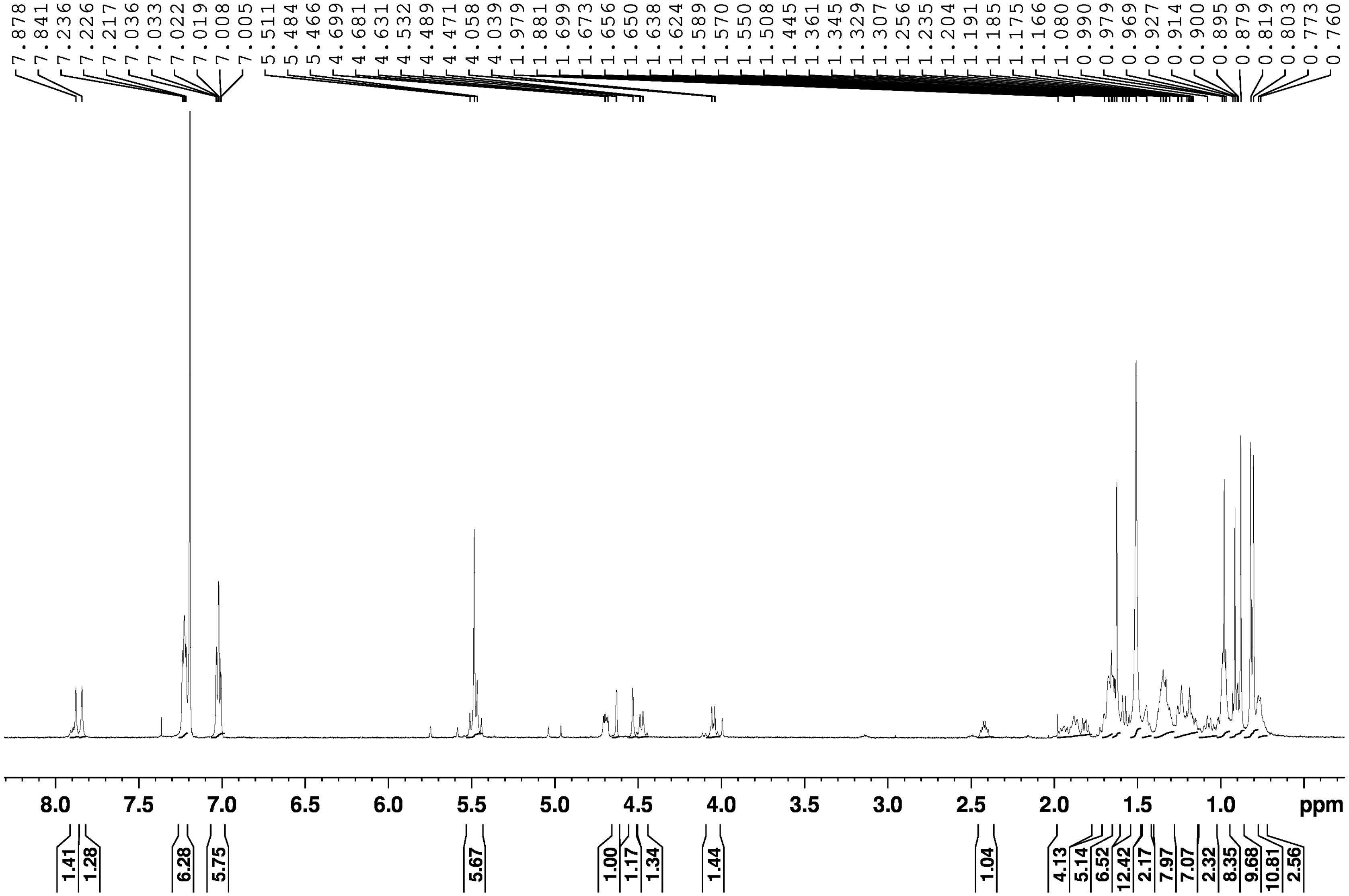

Figure S7 (a): ^{1}H NMR spectrum of 3,28-O,O′-Di{1-(4-fluorobenzyl-1H-[1,2,3]-triazol-4-yl)carbonyl}betulin (**6b**)

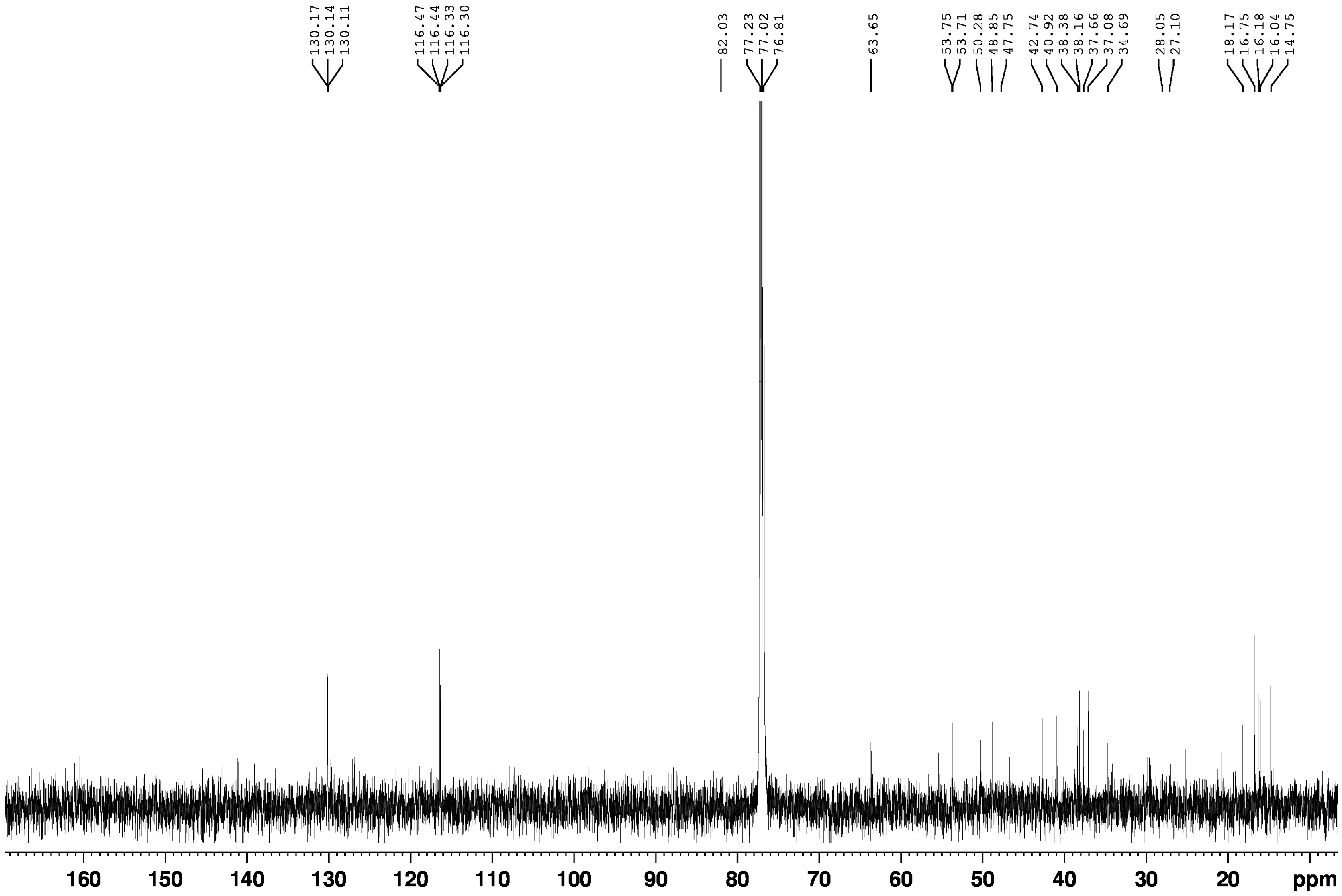

Figure S7 (b): ^{13}C NMR spectrum of 3,28-O,O′-Di{1-(4-fluorobenzyl-1H-[1,2,3]-triazol-4-yl)carbonyl}betulin (**6b**)

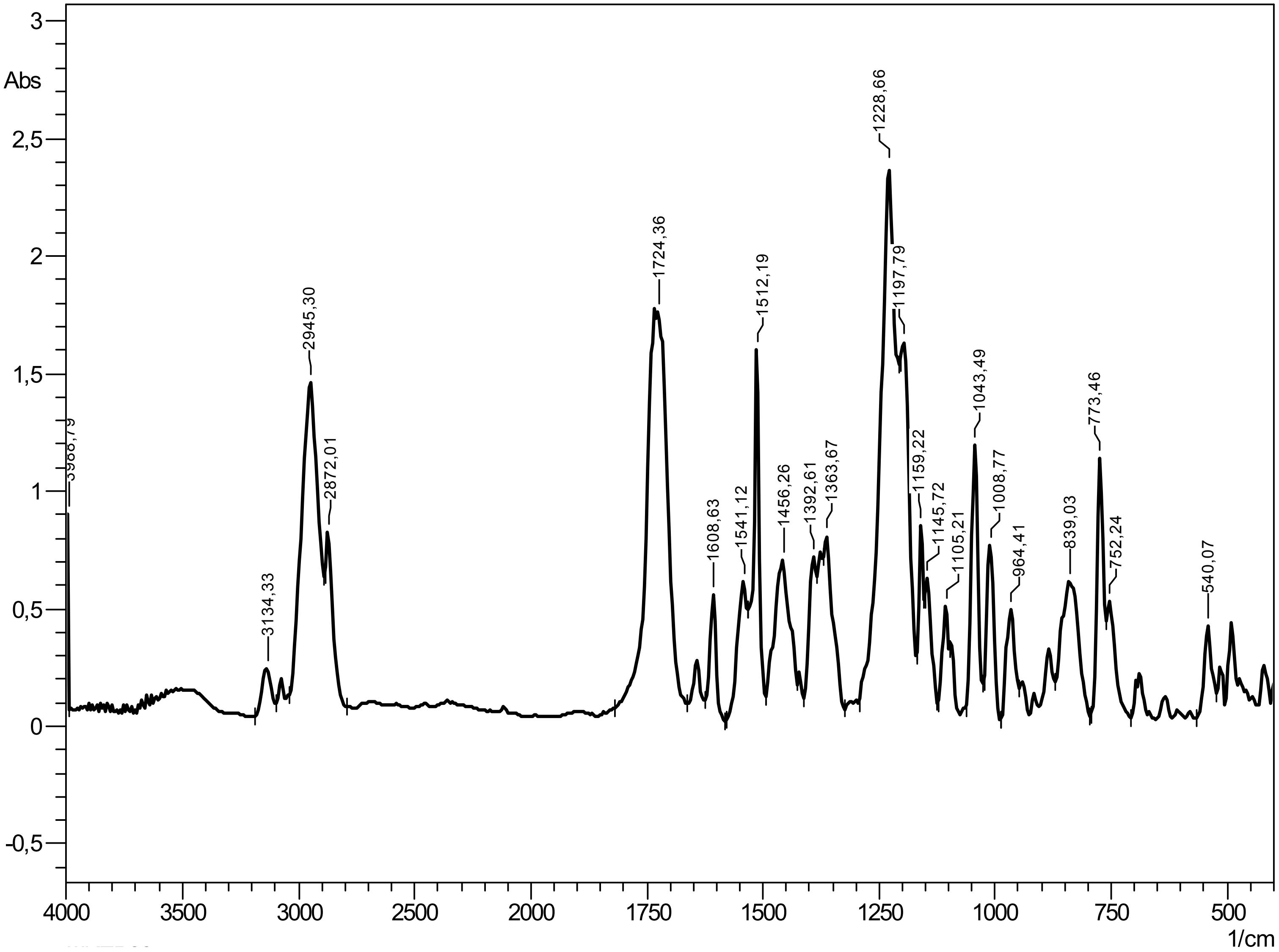

Figure S7 (c): IR spectrum of 3,28-O,O′-Di{1-(4-fluorobenzyl-1H-[1,2,3]-triazol-4-yl)carbonyl}betulin (**6b**)

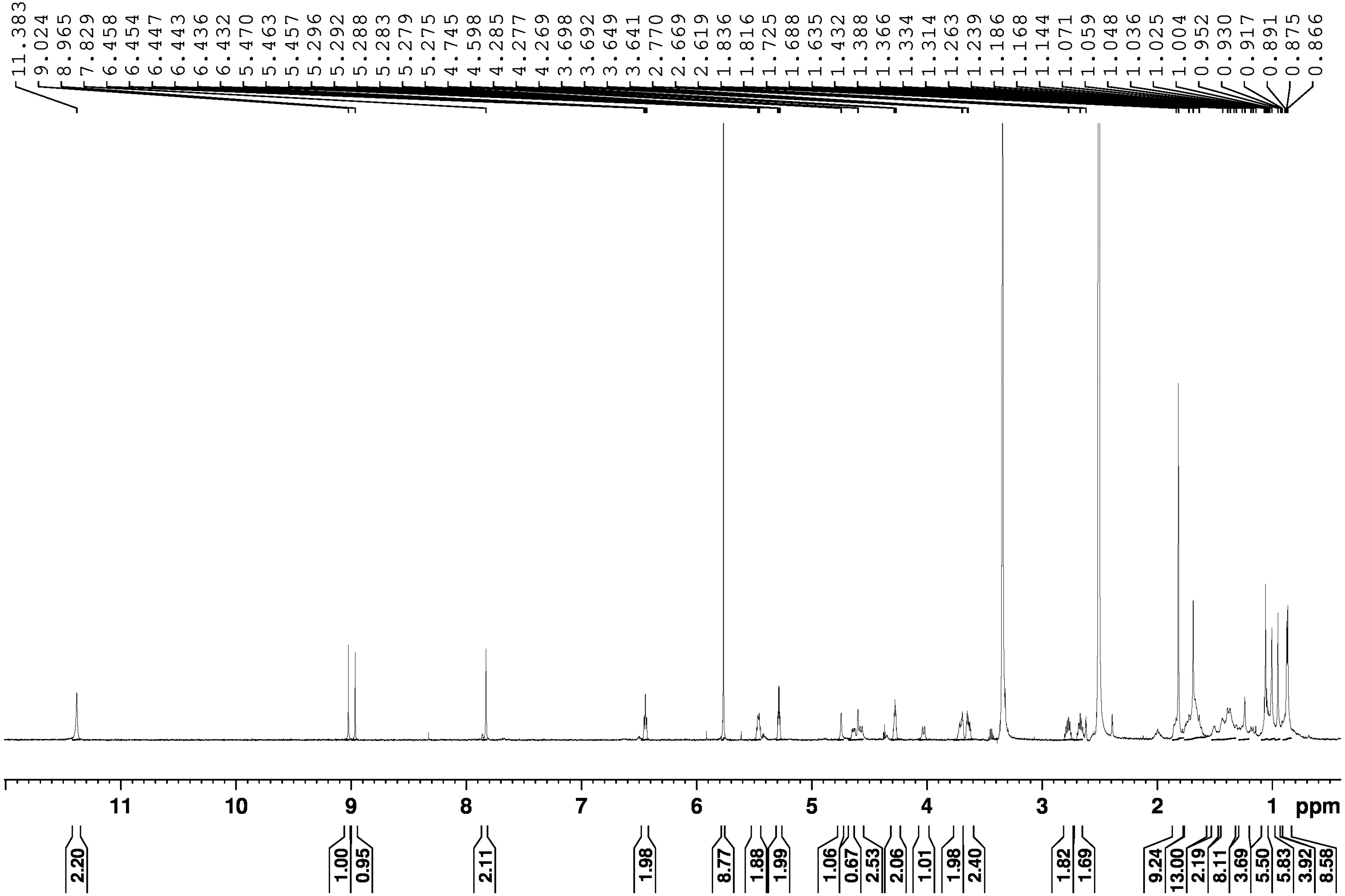

Figure S8 (a): ^{1}H NMR spectrum of 3,28-O,O′-Di{1-(3-hydroxypropyl-1H-[1,2,3]-triazol-4-yl)carbonyl}betulin (**6h**)

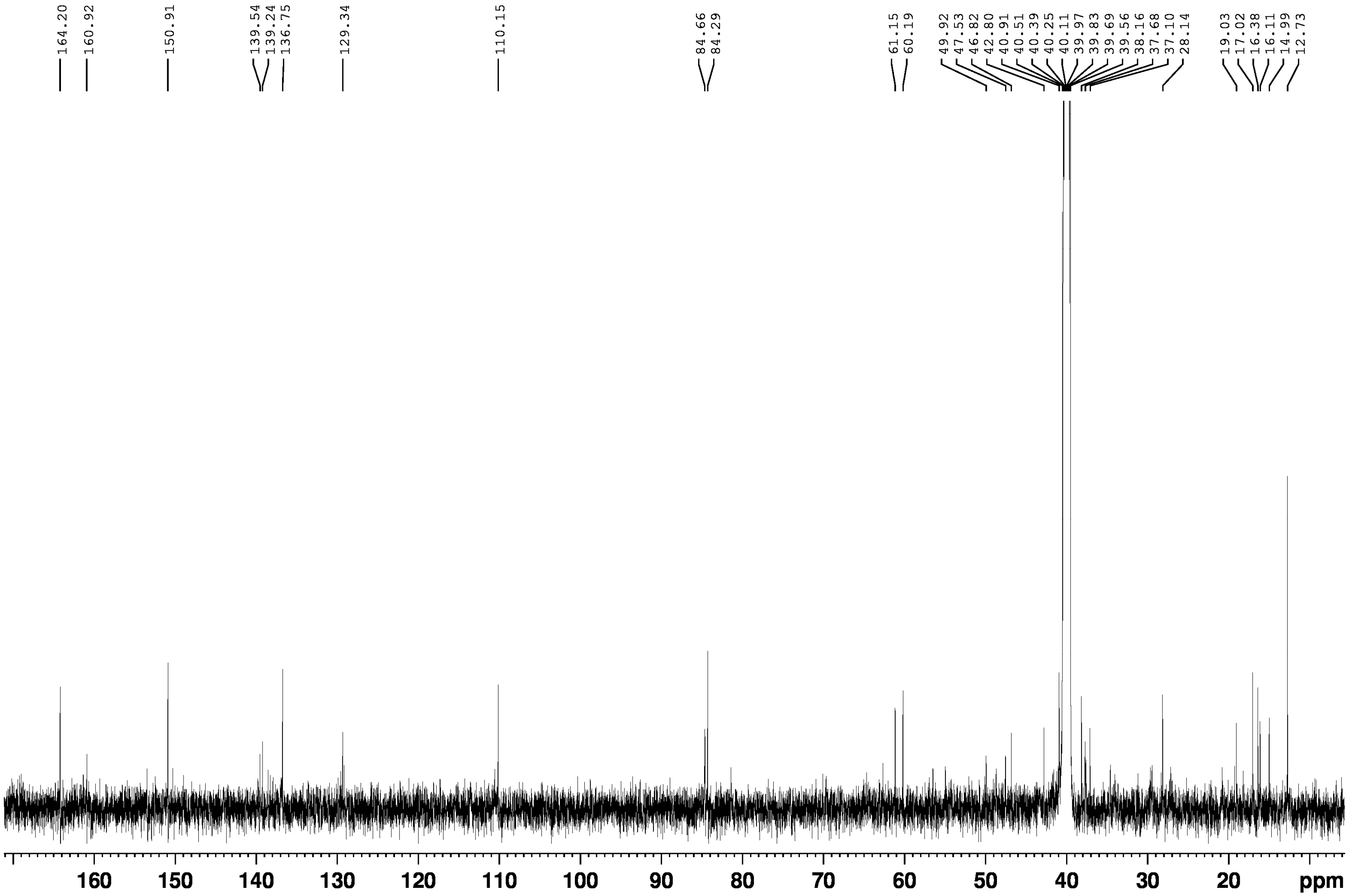

Figure S8 (b): ^{13}C NMR spectrum of 3,28-O,O′-Di{1-(3-hydroxypropyl-1H-[1,2,3]-triazol-4-yl)carbonyl}betulin (**6h**)

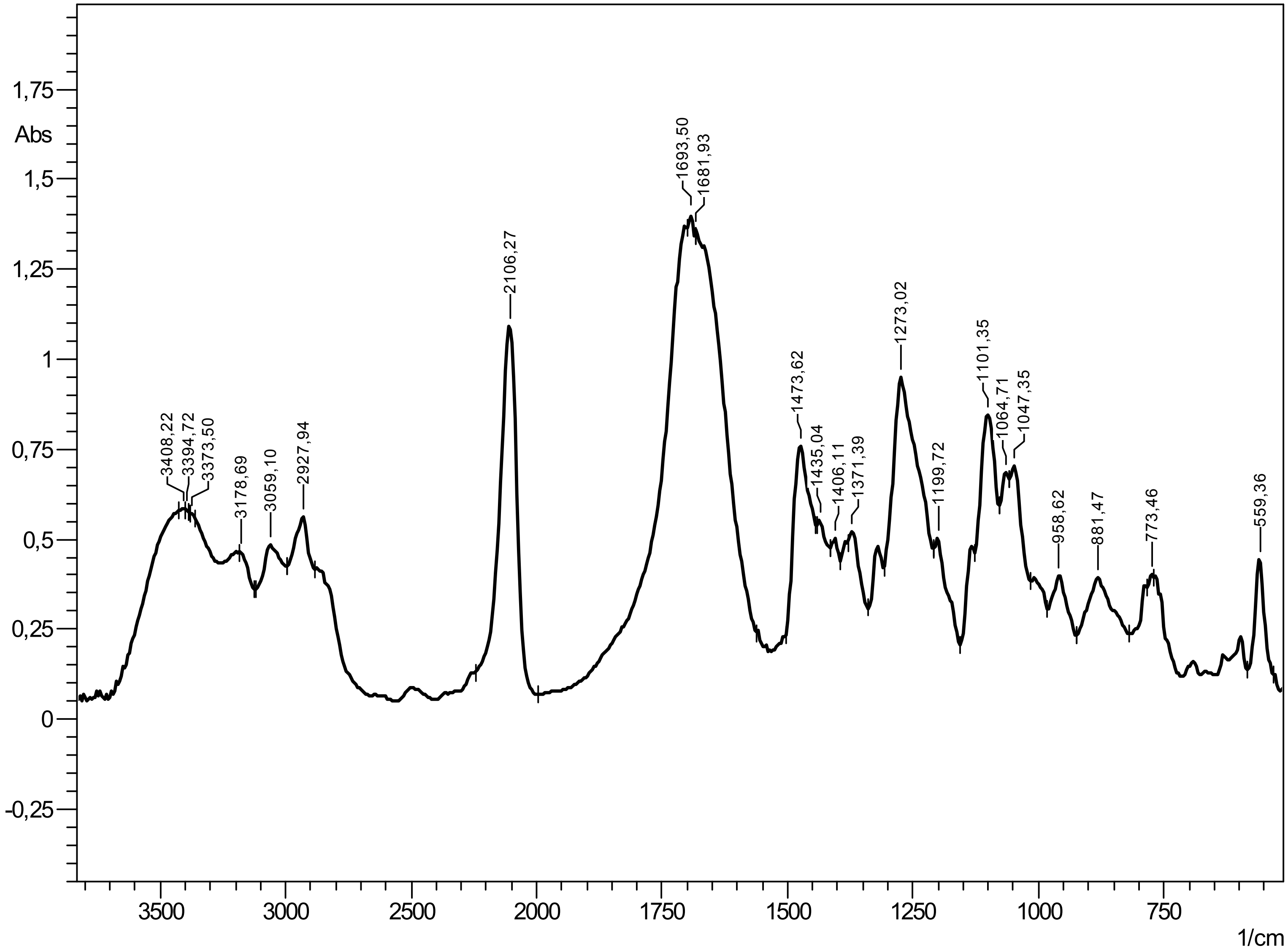

Figure S8 (c): IR spectrum of 3,28-O,O′-Di{1-(3-hydroxypropyl-1H-[1,2,3]-triazol-4-yl)carbonyl}betulin (**6h**)
